# Chromosome-scale genome assembly of kiwifruit *Actinidia eriantha* with single-molecule sequencing and chromatin interaction mapping

**DOI:** 10.1093/gigascience/giz027

**Published:** 2019-04-03

**Authors:** Wei Tang, Xuepeng Sun, Junyang Yue, Xiaofeng Tang, Chen Jiao, Ying Yang, Xiangli Niu, Min Miao, Danfeng Zhang, Shengxiong Huang, Wei Shi, Mingzhang Li, Congbing Fang, Zhangjun Fei, Yongsheng Liu

**Affiliations:** 1 School of Horticulture, Anhui Agricultural University, 130 Chang Jiang Xi Lu, Hefei, Anhui 230036, China; 2 Ministry of Education Key Laboratory for Bio-resource and Eco-environment, College of Life Science, State Key Laboratory of Hydraulics and Mountain River Engineering, 29 Wang Jiang Lu, Sichuan University, Chengdu, Sichuan 610064, China; 3 School of Food Science and Engineering, Hefei University of Technology, 193 Tun Xi Lu, Hefei, Anhui 230009, China; 4 Boyce Thompson Institute, Cornell University, 533 Tower Road, Ithaca, NY 14853, USA; 5 Sichuan Academy of Natural Resource Sciences, 24 Yi Huan Lu Nan Er Duan, Chengdu, Sichuan 610015, China; 6 U.S. Department of Agriculture–Agricultural Research Service, Robert W. Holley Center for Agriculture and Health, 538 Tower Road, Ithaca, NY 14853, USA

**Keywords:** kiwifruit, *Actinidia eriantha*, Genome assembly, single molecular sequencing, high-throughput chromosome conformation capture

## Abstract

**Background:**

Kiwifruit (*Actinidia* spp.) is a dioecious plant with fruits containing abundant vitamin C and minerals. A handful of kiwifruit species have been domesticated, among which *Actinidiaeriantha* is increasingly favored in breeding owing to its superior commercial traits. Recently, elite cultivars from *A. eriantha* have been successfully selected and further studies on their biology and breeding potential require genomic information, which is currently unavailable.

**Findings:**

We assembled a chromosome-scale genome sequence of *A. eriantha* cultivar White using single-molecular sequencing and chromatin interaction map–based scaffolding. The assembly has a total size of 690.6 megabases and an N50 of 21.7 megabases. Approximately 99% of the assembly were in 29 pseudomolecules corresponding to the 29 kiwifruit chromosomes. Forty-three percent of the *A. eriantha* genome are repetitive sequences, and the non-repetitive part encodes 42,988 protein-coding genes, of which 39,075 have homologues from other plant species or protein domains. The divergence time between *A. eriantha* and its close relative *Actinidia chinensis* is estimated to be 3.3 million years, and after diversification, 1,727 and 1,506 gene families are expanded and contracted in *A. eriantha*, respectively.

**Conclusions:**

We provide a high-quality reference genome for kiwifruit *A*. *eriantha*. This chromosome-scale genome assembly is substantially better than 2 published kiwifruit assemblies from *A*. *chinensis* in terms of genome contiguity and completeness. The availability of *the A*. *eriantha* genome provides a valuable resource for facilitating kiwifruit breeding and studies of kiwifruit biology.

## Introduction

Kiwifruit is often referred to as the king of fruits owing to its remarkably high vitamin C content and abundant minerals [1, 2]. Native to China, kiwifruit belongs to the genus *Actinidia*, which contains 54 species and 75 taxa [3]. All species in this genus are perennial, deciduous, and dioecious plants with a climbing or scrambling growth habit, and they also have many common morphological features including the characteristic radiating arrangement of styles of the female flower and the structure of the fruit [4]. Despite rich germplasm resources in kiwifruit, only a few *Actinidia* species have been domesticated, such as *Actinidia chinensis* var. chinensis, *A. chinensis* var. deliciosa, and *Actinidia eriantha*, whose fruit size are close to commercial standard [5–7].

**Figure 1:**
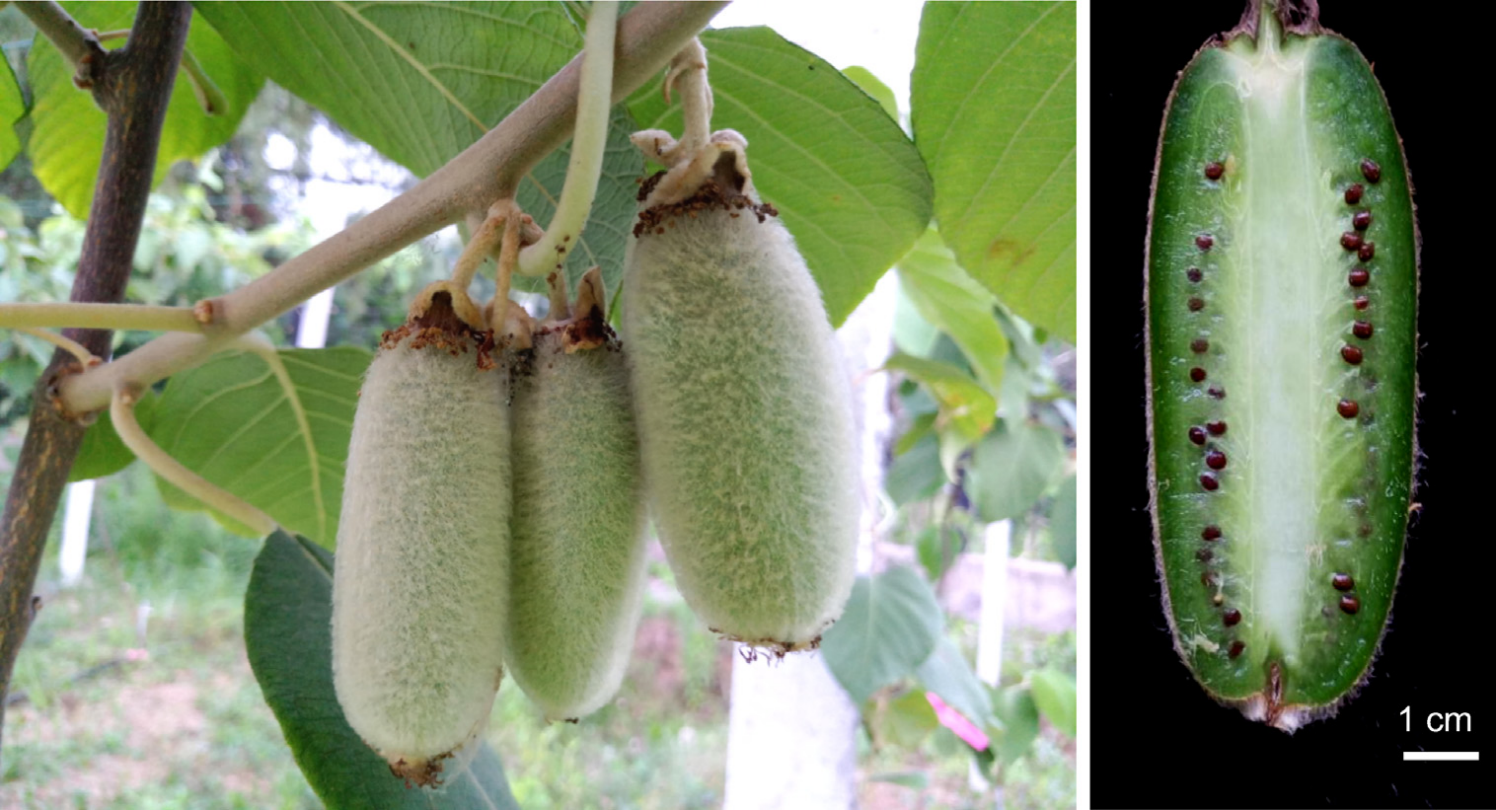
Tree and fruits of *A. eriantha* cv. White.

Owing to its strong resistance to *Pseudomonas syringae* pathovar*Actinidiae*, long shelf-life, enriched ascorbic acid, and peelable skin [7–11], *A. eriantha* (2n = 58) has been favored in kiwifruit breeding. Recently, new cultivars have been selected either from the wild germplasm of *A. eriantha* such as “White” (Fig. 1) or from the interspecific hybridization between *A*. *eriantha* (male) and *A*. *chinensis* (female) such as “Jinyan” [7, 12]. White has particularly large fruits (mean, 96 g) with green flesh and favorable flavor and has been widely cultivated in China [7].

*Actinidia eriantha* (*Actinidia eriantha*, NCBI:txid165200) has also been used for genetic and genomic studies thanks to its high efficiency in genetic transformation and relatively short phase of juvenility [13]. The flowering and fruiting of *A. eriantha* can be accomplished within 2 years in greenhouse conditions with a low requirement for winter chilling [13]. In addition, the roots of *A. eriantha*, which contain many bioactive compounds such as triterpenes and polysaccharides, are used as a traditional Chinese medicine for the treatment of gastric carcinoma, nasopharyngeal carcinoma, breast carcinoma, and hepatitis [12, 14].

Previously, 2 kiwifruit genomes were published and both were varieties of *A. chinensis* (“Hongyang” and “Red5”) [15, 16]. These short-read–based assemblies are very fragmented, possibly due to the high complexity and heterozygosity of the kiwifruit genomes, as well as technical limitations. Here, we used single-molecular sequencing combined with high-throughput chromosome conformation capture (Hi-C) technology to assemble the genome of the elite kiwifruit cultivar “White” of *A. eriantha*. The availability of this high-quality chromosome-scale genome sequence not only provides fundamental knowledge regarding kiwifruit biology but also presents a valuable resource for kiwifruit breeding programs.

### Sample collection and genome sequencing

Fresh young leaves were collected from a female individual of *A*. *eriantha* cv. White. High molecular weight genomic DNA was extracted using the CTAB (cetyl trimethylammonium bromide) method as described in the protocol [17]. To construct genomic libraries (SMRTbell libraries) for Pacific Biosciences (PacBio) long-read sequencing, high molecular weight genomic DNA was sheared into fragments of ∼20 kilobases (kb) using a Covaris g-Tube (KBiosciences part No. 520079), enzymatically repaired, and converted to SMRTbell template following the manufacturer's instructions (DNA Template Prep Kit 1.0, PacBio part No. 100-259-100). The templates were size-selected using a BluePippin (Sage Science, Inc., Beverly, MA, USA) to enrich large DNA fragments (>10 kb) and then sequenced on a PacBio Sequel system. A total of 9 single-molecule real-time (SMRT) cells were sequenced, yielding 3,889,480 million reads with a mean and median length of 10,065 and 15,661 base pairs (bp), respectively, and a total of 39.1 gigabase (Gb) sequences, ∼52.5× coverage of the kiwifruit genome with an estimated size of 745.3 megabases (Mb) based on the flow cytometry analysis (Fig. S1; Table S1).

Three paired-end Illumina libraries with insert sizes of 180, 220, and 500 bp and 7 mate-pair libraries with insert sizes of 3, 4, 5, 8, 10, 15, 17 kb were prepared using Illumina's Genomic DNA Sample Preparation kit and the Nextera Mate Pair Sample Preparation kit (Illumina, San Diego, CA), respectively. All libraries were sequenced on an Illumina HiSeq 2500 system, which yielded ∼80.1 and ∼97.3 Gb of raw sequence data for paired-end and mate-pair libraries, respectively (Table S1). The raw Illumina paired-end reads were processed to remove duplications, adaptors, and low-quality bases using Super-Deduper [18] and Trimmomatic (Trimmomatic, RRID:SCR_011848) [19] (v0.35), and the mate-pair reads were cleaned using NextClip (NextClip, RRID:SCR_005465) [20] (v1.3.1) with default parameters. Finally, we obtained 76.6 and 46.2 Gb high-quality cleaned sequences for paired-end and mate-pair libraries, respectively (Table S1).

To construct the Hi-C library, White plants were grown in a greenhouse, and ∼4–6 g young leaves were then harvested and subsequently fixed in formaldehyde (1% volume/volume [v/v]) for 10 min at room temperature. The fixation was terminated by adding glycine to a final concentration of 0.125 M. The fixed samples were ground into powder in liquid nitrogen and then lysed with the addition of Triton X-100 to a concentration of 1% (v/v). The nuclei were isolated and prepared for Hi-C library construction according to a previously published protocol [21].

### Transcriptome sequencing

To improve gene prediction, we generated transcriptome sequences from a pool of mixed tissues of White including root, stem, leaf, flower, and fruits at 7, 30, 60, 90, and 120 days after anthesis. Total RNA was extracted from these tissues using an RNA extraction kit (BIOFIT, Chengdu, Sichuan, China), treated with DNase I and further purified with RNA clean kit (Promega, Madison, WI, USA). RNA sequencing (RNA-Seq) libraries were constructed with the NEBNext Ultra RNA Library Prep Kit (Illumina, USA), and sequenced on an Illumina HiSeq 2500 system using paired-end mode. A total of ∼19.5 million raw read pairs were obtained, which were processed with Trimmomatic to remove adaptors. The cleaned reads were assembled *de novo* with Trinity (Trinity, RRID:SCR_013048) [22] (v2.4.0). Additionally, we also generated genome-guided assemblies with both Trinity and StringTie (StringTie, RRID:SCR_016323) [23]. Different transcriptome assemblies were eventually integrated by PASA (PASA, RRID:SCR_014656) [24] (v2.3.3) and used as transcript evidence during gene prediction process. Mapping of RNA-Seq reads to the genome assembly was performed with STAR (STAR, RRID:SCR_015899) [25] (v02,0201), and read counting on the coding regions was performed with HTSeq (HTSeq, RRID:SCR_005514) [26] (v0.6.0).

### Chromosome-scale assembly of the *A. eriantha* genome

*Actinidia eriantha* is a diecious plant with a heterozygous diploid genome. We estimated the heterozygosity level through the k-mer spectrum analysis with GenomeScope [27] using sequences from the paired-end library with an insert size of 180 bp. The depth distribution of the derived 17-mers clearly showed 2 separate peaks, based on which we estimated the heterozygosity level of the *A. eriantha* cv. White genome to be 1.21% (Fig. S1).

We then estimated the genome size of *A*. *eriantha* cv. White using flow cytometry analysis, with tomato (*Solanum lycopersicum* cv. Ailsa Craig) used as the reference. We also performed flow cytometry analysis on *A. chinensis* cv. Hongyang. Approximately 1 g of young leaves were washed twice in distilled water and then chopped in ice-cold lysis buffer A (10 mmol/L MgSO_4_, 50 mmol/L KCl, 3.5 mmol/L HEPES [4-(2-hydroxyethyl)-1-piperazineethanesulfonic acid] pH 7.5, 0.3% [v/v] Triton x-100, 2% polyvinylpyrrolidone 30 weight by volume). After 5 min, the crude lysate was passed through a 75-μm pore size nylon mesh to remove large cellular debris. The filtrate (1 mL) was transferred to a 1.5-mL plastic tube and centrifuged at 1000 rpm for 5 min. The supernatant was discarded, and the nuclei were then resuspended with lysis buffer B (10 mmol/L MgSO_4_, 50 mmol/L KCl, 3.5 mmol/L HEPES pH 7.5, 0.3% [v/v] Triton x-100, 0.4 mg/mL propidium iodide, 0.04 mg/mL RNase). After 15 min, samples were analyzed using a FACS Vantage SE flow cytometer (Becton-Dickinson, San José, USA). Four biological replicates were performed. Based on the 950-Mb genome of tomato, the genome size of White was estimated to be 745.3 ± 7.9 Mb, similar to the genome size of *A. chinensis* (Fig. S1) and consistent with that in a previous report (758 Mb; [28]).

We used a strategy that took into account the unique advantage of different assemblers to construct the White genome using PacBio long reads. First, PacBio long reads were corrected and assembled using the Canu program (Canu, RRID:SCR_015880) [29] (v1.7), which is a modularized pipeline consisting of 3 primary stages—read correction, trimming, and assembly. The Canu-corrected reads were also assembled independently with the wtdbg program [30], a fast assembler for long noisy reads. Subsequently, the 2 independent assemblies (one with Canu and another with wtdbg) were merged by Quickmerge [31] (v0.2) to improve the contiguity. The merged assembly was further processed to correct errors using Pilon (Pilon, RRID:SCR_014731) [32] (v1.22) with high-quality cleaned Illumina reads from all paired-end and mate-pair libraries, representing a total genome coverage of 171× (Table S1). This yielded 2,818,370 nucleotides, 2,495,388 insertions, and 1,691,495 deletions being corrected. The resulting final assembled *A*. *eriantha* cv. White genome contained 4,076 contigs with an N50 length of 539,246 bp and a cumulative size of 690,376,929 bp (Table 1). The contiguity and completeness of this assembly far exceeds that of 2 published kiwifruit *A. chinensis* genomes (Table 1).

**Table 1:** Assembly statistics

Parameter	*A. eriantha*	*A. chinensis*	
	White	Hongyang	Red5
**Contigs**			
Total contig No.	4,076	26,721	39,868
Total contig length (Mb)	690.4	604.2	
Contig N50 (kb)	539.2	58.9	
Contig N90 (kb)	50.7	11.6	
Longest contig length (kb)	3,260.20	423.5	
**Scaffolds**			
Total scaffold No.	1,735	7,698	3,887
Total scaffold length (Mb)	690.6	616.1	550.5
Scaffold N50 (kb)	23,583.9	646.8	623.8
Scaffold N90 (kb)	20,112.1	122.7	140.7
Longest scaffold length (Mb)	28.6	3.4	4.43
Anchored to chromosome (Mb/%)	682.4/98.84	452.4/73.4	547.9/98.9
Anchored with order and orientation (Mb/%)	634.4/91.90	333.6/54.1	

**Figure 2:**
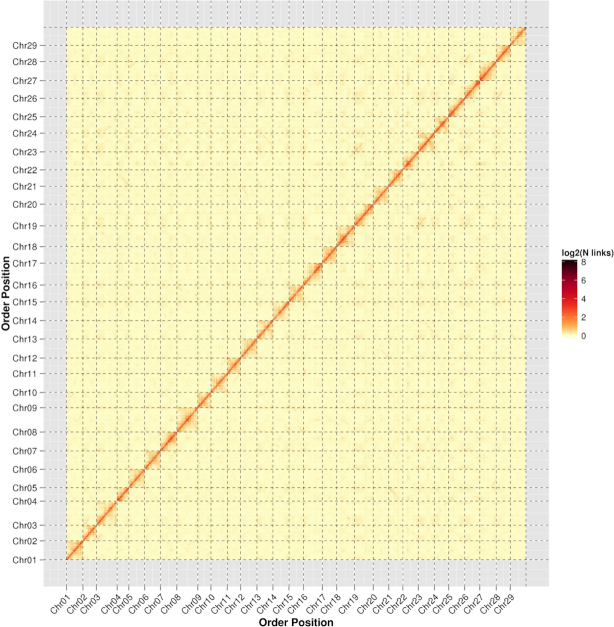
Chromatin interaction map of *A. eriantha* derived from Hi-C data. Each group represents an individual chromosome.

**Figure 3:**
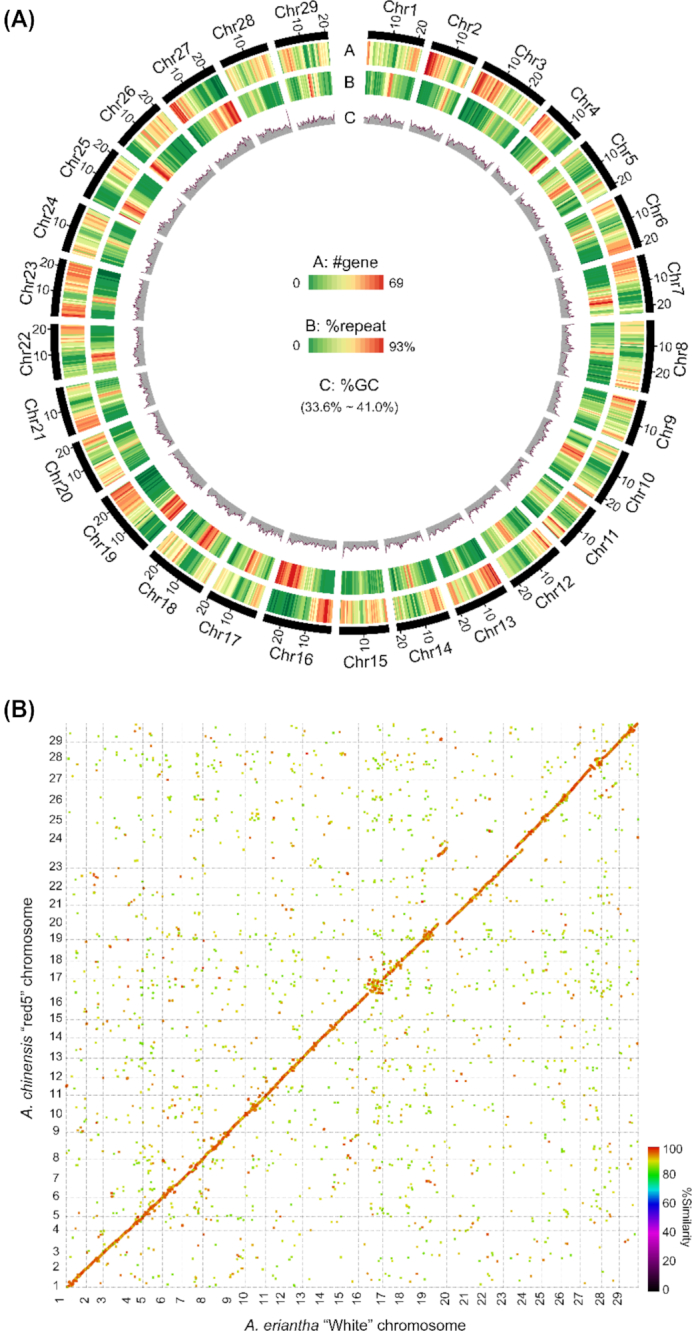
Genome of *A*. *eriantha* and synteny between the 2 kiwifruit species. (a) Genome landscape of *A. eriantha* cv. White. Track A: gene density, Track B: repeat density, Track C: guanine or cytosine (GC) content; all were calculated in a 500-kb window. (b) Genome synteny between *A. eriantha* cv. White and *A. chinensis* cv. Red5.

To scaffold the contigs on the basis of chromatin interaction maps inferred from the Hi-C data, we first used HiC-Pro [33] to evaluate and filter the cleaned Hi-C reads. The Hi-C data usually contain a considerable part of invalid interaction read pairs, which are non-informative and need to be filtered out beforehand. Among the 51 million read pairs that were uniquely aligned to the *A*. *eriantha* assembly, 33 million (64.1%) were valid interaction pairs and their insertion size spanned predominantly from dozens to hundreds of kilobases, therefore providing efficient information for scaffolding. As a part of error correction of the assembly, we used valid Hi-C reads to identify misassembled contigs. In principle, a genuine contig should display a continuous Hi-C interaction map whereas the discrete distribution of an interaction map likely indicates a misassembly. We examined the interaction map for each contig and broke 51 that were possibly misassembled. Subsequently, the corrected PacBio assembly was used for scaffolding with the LACHESIS program [34] and parameters “CLUSTER_MIN_RE_SITES = 48, CLUSTER_MAX_LINK_DENSITY = 2, CLUSTER_NONINFORMATIVE_RATIO = 2, ORDER_MIN_N_RES_IN_TRUN = 14, ORDER_MIN_N_RES_IN_SHREDS = 15.” LACHESIS assigned 3,666 contigs with a total size of 682,355,494 bp (98.84% of the assembly) into 29 groups corresponding to the 29 kiwifruit chromosomes (Figs. 2 and 3a), among which 634,430,648 bp (91.90%) had defined order and orientation (Table 1 and Table S2). The final chromosome-scale assembly had a total length of 690,781,529 bp and an N50 of 23,583,865 bp.

### Evaluation of the genome assembly

We first evaluated the quality of the assembled *A*. *eriantha* cv. White genome by mapping Illumina genomic and RNA-Seq reads to the assembly. Reads from the paired-end genomic library (with insert size of 180 bp) had a very high mapping rate (98.7%), and the properly paired read mapping rate was 92.0%. For the RNA-Seq reads, 91.7% could be mapped to the genome and 87.1% were uniquely mapped. The high mapping ratio of both genomic and RNA-Seq reads suggests a high quality of the *A*. *eriantha* cv. White assembly.

We then identified synteny between the *A*. *eriantha* cv. White assembly and the assembly of *A. chinensis* cv. Red5 using MUMMER [35] (v4.0.0beta2). In general, the 2 assemblies showed a high macro-collinearity, with only a few inconsistencies (Fig. 3b). A detailed check of the major inconsistent regions using genetic maps [36] and mate-pair read alignments confirmed the high quality of the *A*. *eriantha* cv. White genome assembly and particularly enabled us to discover that in the Red5 genome a ∼8-Mb region was possibly misassembled into chromosome 23 (Fig. S2).

### Repeat annotation

Repeats were annotated following a protocol described in Campbell et al. [37]. The customized repeat library was built to include both known and novel repeat families. We first searched the assembly for miniature inverted transposable elements (MITEs) using MITE-Hunter [38] with default parameters. The long terminal repeat (LTR) retrotransposons were then identified from the *A*. *eriantha* cv. White genome using LTRharvest and LTRdigest wrapped in the GenomeTools package [39]. The LTR identification pipeline was run iteratively to collect both recent (sequence similarity ≥99%) and old (sequence similarity ≥85%) LTR retrotransposons. Candidates from each run were filtered on the basis of the elements typically encoded by LTR retrotransposons. The default parameters (–minlenltr 100 –maxlenltr 6000 –mindistltr 1500 –maxdistltr 25,000 –mintsd 5 –maxtsd 5 –motif tgca) were used in LTR calling according to Campbell et al. [37]. An initial repeat masking of *A*. *eriantha* cv. White genome was performed with the repeat library derived by combining the identified MITEs and LTR transposons. The repeat-masked genome was fed to RepeatModeler (RepeatModeler, RRID:SCR_015027) [40] to identify novel repeat families. Finally, all identified repeat sequences were combined and searched against a plant protein database from which transposon encoding proteins were excluded. Elements with significant similarity to plant genes were removed. The final repeat library contained 1,670 families, and 526 of them were potentially novel repeat families. We used this species-specific repeat library to mask the *A*. *eriantha* cv. White genome. Approximately 43.3% of the *A*. *eriantha* cv. White genome was masked, and the largest family of repeats was LTR transposons (Table S3). Repeat content identified in *A*. *eriantha* cv. White was much higher than that in *A*. *chinensis* (e.g., 36% in Hongyang [15]), and this difference may be largely due to the improvement of the repeat region assembly with PacBio long reads. In addition, divergence between the 2 kiwifruit species could also contribute to this difference.

### Prediction and functional annotation of protein-coding genes

Protein-coding genes were predicted from the repeat-masked *A*. *eriantha* cv. White genome with the MAKER-P program [37] (v2.31.10), which integrates evidence from protein homology, transcripts, and *ab initio* predictions. The homology-based evidence was derived by aligning proteomes from 20 plant species to the White genome assembly with Exonerate (Exonerate, RRID:SCR_016088) (v2.26.1) [41]. SNAP [42], AUGUSTUS (Augustus, RRID:SCR_008417) [43] (v3.3), and GeneMark-ES (GeneMark, RRID:SCR_011930) [44] (v4.35) were used for *ab initio* gene predictions. RNA-Seq data generated in this study were assembled and the assembled contigs were aligned to the White genome assembly to provide transcript evidence. Predictions supported by the 3 different sources of evidence were finally integrated by MAKER-P (MAKER, RRID:SCR_005309), which resulted in a total of 52,514 primitive gene models. We then filtered and polished these gene models by 2 steps. First, we combined our RNA-Seq data with others collected from a previous study [45], and mapped the reads to the White genome using the STAR program [25], and a total of 266 million read pairs were mapped. Based on the mapping, a raw count for each predicted gene model was derived and then normalized to CPM (counts per million mapped read pairs). Gene models with ultra-low expression (CPM < 0.1) were less likely to be real genes. Furthermore, we found that these genes with ultra-low expression had relatively high annotation edit distance score, an indication of low confidence as defined by the MAKER-P program. Therefore, for gene models with CPM < 0.1, we only kept those containing both pfam domains and homologous sequences in the NCBI non-redundant protein database. After this filtering process 42,751 gene models were kept. Second, the predicted protein-coding genes of kiwifruit *A*. *chinensis* cv. Red5 have been manually curated [16], and therefore these gene models should have relatively higher accuracy and could be used to modify *A*. *eriantha* cv. White gene models whose predictions were not consistently supported by the different types of evidence. To this end, we performed another 2 *ab initio* predictions using BRAKER [46] and GeMoMa [47] (v1.5.2) with Red5 proteome as the sole evidence. These 2 predictions were compared with the gene models predicted by MAKER-P. Consequently, a total of 237 gene models not predicted by MAKER-P were added and another 415 gene models that had better predictions by BRAKER2 or GeMoMa were used to replace the corresponding gene models predicted by MAKER-P. Finally, we obtained a total of 42,988 protein-coding genes in the *A*. *eriantha* cv. White genome, with a mean coding sequence size of 1,004 bp and containing a mean of 5 exons.

The predicted genes were functionally annotated by blasting their protein sequences against TAIR (TAIR, RRID:SCR_004618) [48], Swiss-Prot [49], and TrEMBL [50] databases with an E-value cutoff of 1E–5. Functional descriptions of the protein hits were assembled with the AHRD program [51] and transferred to *A*. *eriantha* genes. Protein domains were identified using InterProScan (InterProScan, RRID:SCR_005829) [52] (v5.29–68.0) by searching the protein sequences against domain databases including PANTHER (PANTHER, RRID:SCR_004869) [53], Pfam (Pfam, RRID:SCR_004726) [54], SMART (SMART, RRID:SCR_005026) [55], and PROSITE (PROSITE, RRID:SCR_003457) [56]. The gene ontology terms were assigned to the *A*. *eriantha* cv. White predicted genes using the Blast2GO program (Blast2GO, RRID:SCR_005828) [57] with entries from the NCBI protein database and InterProScan. Collectively, 90.9% (*n* = 39,075) of the predicted genes contain ≥1 annotation from the above databases (Table S4).

### Evolutionary and comparative genomic analysis

**Figure 4:**
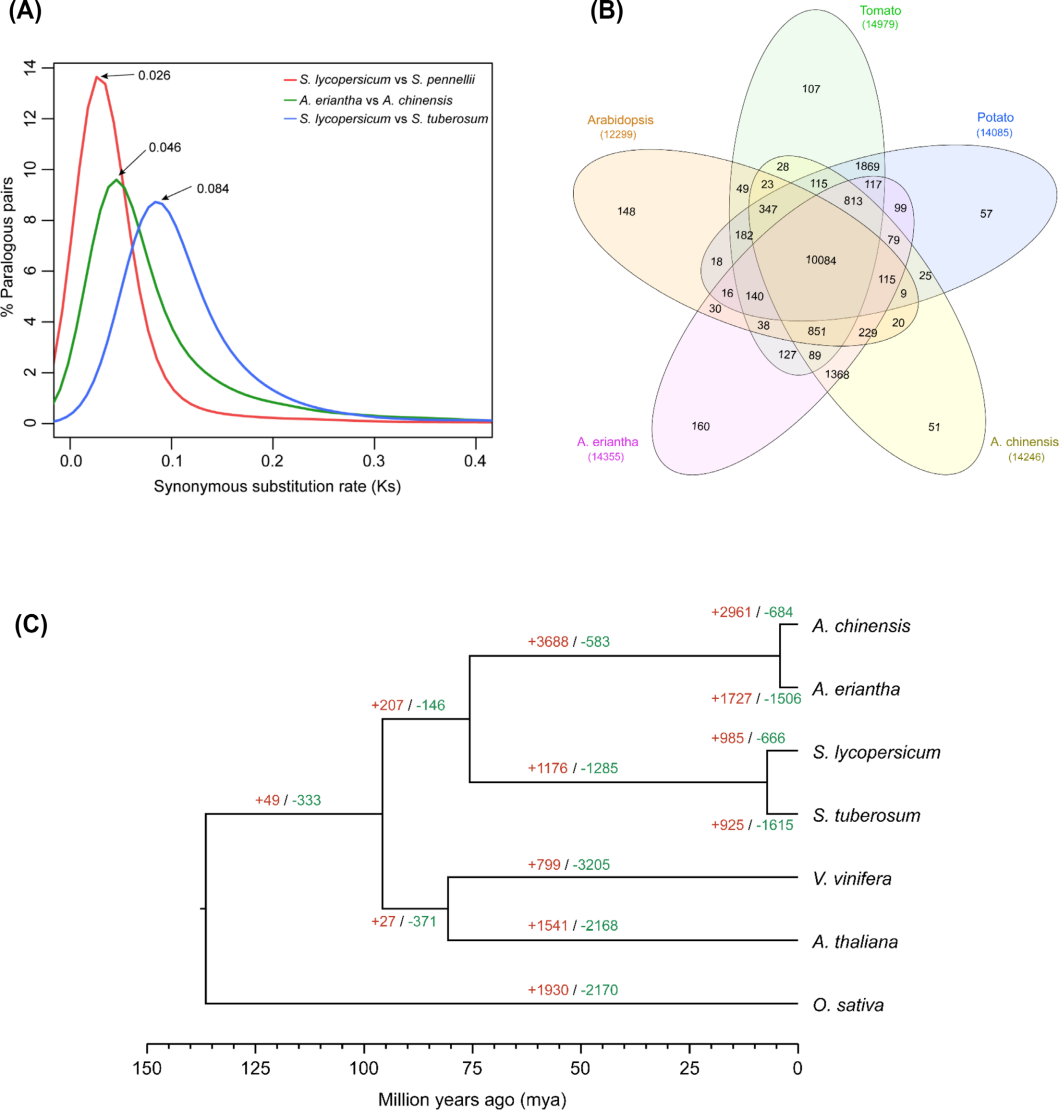
Evolutionary and comparative genomic analyses. (a) Distribution of synonymous substitution rate (Ks) between *A. eriantha* and *A. chinensis*, *S. lycopersicum* and *S. pennellii*, and *S. lycopersicum* and *S. tuberosum*. (b) Orthogroups shared by selected species. (c) Species phylogenetic tree and gene family evolution. Numbers on the branch indicate counts of gene families that are under either expansion (red) or contraction (green).

To infer the divergence time between *A. eriantha* and *A. chinensis*, we identified gene orthology between the 2 species using MCScanX [58] and calculated the synonymous substitution rate (Ks) between each orthologous pair. Three additional species, cultivated tomato (*Solanum lycopersicum*), wild tomato (*Solanum pennellii*), and potato (*Solanum tuberosum*), were also included in the analysis. The Ks distribution (Fig. 4a) suggested that the divergence between the 2 kiwifruit species was earlier than that between the 2 tomato species. We dated the divergence by assuming a strict molecular clock [59], and the time when *A. eriantha* and *A. chinensis* separated was estimated to be ∼3.3 million years ago (Mya), compared to ∼1.9 Mya between *S. lycopersicum* and *S. pennellii* and ∼6.0 Mya between *S. lycopersicum* and *S. tuberosum*. Gene family evolution was analyzed by comparing genomes of *A. eriantha*, *A. chinensis*, *S. lycopersicum*, *S. tuberosum*, *Vitis vinifera*, *Arabidopsis thaliana*, and *Oryza sativa*. A total of 17,593 orthogroups were defined by OrthoFinder [60] (v2.2.6), among which 1,246 were single-copy gene families (Fig. 4b). The single-copy family genes were aligned and concatenated to build a species phylogenetic tree using IQ-TREE [61] (v1.5.5) with a best-fitting model (Fig. 4c). Gene family expansion/contraction along the branches of the phylogenic tree was analyzed by CAFÉ [62] (v4.1). Finally, a total of 1,727 and 1,506 gene families were found apparently expanded and contracted, respectively, in *A. eriantha* (Fig. 4c).

## Conclusion

Herein, we report a high-quality reference genome of kiwifruit *A*. *eriantha* cv. White. The assembly from single-molecular sequencing combined with Hi-C scaffolding yielded a more highly continuous and complete genome than the 2 previously published kiwifruit genomes. This genome will provide a valuable source for exploration of the genetic basis of unique traits in kiwifruit and also facilitate studies of sexual determination loci in the dioecious plants.

## Availability of supporting data and materials

This Whole Genome Shotgun project has been deposited at DBJ/ENA/GenBank under the accession No. QOVS00000000. The version described in this paper is version QOVS01000000. Raw sequencing reads have been deposited in the Sequence Read Archive database under the accession No. SRP155011. The *Actinidia eriantha* cv. White genome sequence and the annotation are also available via the *GigaScience* database, GigaDB [63]. Detailed protocols of computational analyses have been deposited in protocols.io [64].

## Additional files

**Figure S1**. Genome characteristics of *A. eriantha and A. chinensis*. (a) Flow cytometry analyses of *A. eriantha* cv. White and *A. chinensis* cv. Hongyang. The main peak (I) indicates G0/G1 cells and the secondary peak (II) represents G2/M cells. (b) Flow cytometry analyses of *A. eriantha* cv. White and *Solanum lycopersicum* cv. Ailsa Craig. Peaks a and b represent the G0/G1 cells of “White” and “Ailsa Craig”, respectively. The genome size of “White” was estimated to be 745.3 ± 7.9 Mb using “Ailsa Craig” as the reference. (c) 17-mer distribution of “White” genomic reads (180bp paired-end library).

**Figure S2**. Examination of assembly inconsistencies between *A. eriantha* cv. White and *A. chinensis* cv. Red5. (a) Validation of genome assembly of “White” using genetic maps. Horizontal lines within “White” chromosomes indicate gapped regions and lines between chromosomes of 2 assemblies indicate syntenic regions. (b) A chromosomal segment assembled into the Chr23 in *A*. *chinensis* cv. Red5 is syntenic to the region located at the terminus of Chr19 in *A. eriantha* cv. White. (c) Snapshots of Illumina mate-pair reads mapped to the junctions of the break point as well as nearby regions supporting the assembly of “White.”

Supp_Tables.xlsx

## Abbreviations

BLAST: Basic Local Alignment Search Tool; bp: base pair; CPM: counts per million mapped read pairs; CTAB: cetyl trimethylammonium bromide; cv.: cultivar; Gb: gigabase; HEPES: 4-(2-hydroxyethyl)-1-piperazineethanesulfonic acid; Hi-C: high-throughput chromosome conformation capture; LTR: long terminal repeat; Mb: megabase; MITE: miniature inverted transposable element; Mya: million years ago; NCBI: National Center for Biotechnology Information; PacBio: Pacific Biosciences; RNA-Seq: RNA sequencing; SMRT: single-molecule real-time; var.: variety; v/v: volume/volume.

## Competing interests

The authors declare that they have no competing interests.

## Funding

This work was supported by grants from the National Natural Science Foundation of China (31471157 and 31700266), National Foundation for Germplasm Repository of Special Horticultural Crops in Central Mountain Areas of China (NJF2017-69), National Science Fund for Distinguished Young Scholars (30825030), Key Project from the Government of Sichuan Province (2013NZ0014, 2016NZ0105), Key Project from the Government of Anhui Province (2012AKKG0739; 1808085MC57), and the US National Science Foundation (IOS-1339287 and IOS-1539831).

## Authors’ contributions

W.T., J.Y., X.T., Y.Y., X.N., M.M., D.Z., S.H., W.S., C.F., and M.L. collected plant samples, extracted DNA/RNA, and performed transcriptome sequencing and gene expression analyses; W.T., X.S., J.Y., X.T., C.J., Z.F., and Y.L. performed DNA sequencing, genome assembly, gene annotation, evolution and comparative genomic analyses, and website construction; X.S., W.T., Z.F., and Y.L. wrote and revised the manuscript; Y.L. and Z.F. conceived strategies, designed experiments, and managed projects. All authors read and approved the manuscript.

## Supplementary Material

GIGA-D-18-00282_Original-Submission.pdf

GIGA-D-18-00282_Revision-1.pdf

GIGA-D-18-00282_Revision-2.pdf

GIGA-D-18-00282_Revision_3.pdf

GIGA-D-18-00282_Revision_4.pdf

Response_to_Reviewer_Comments_Original_Submission.pdf

Response_to_Reviewer_Comments_Revision_1.pdf

Response_to_Reviewer_Comments_Revision_2.pdf

Response_to_Reviewer_Comments_Revision_3.pdf

Reviewer_1_Report_Original_Submission -- Robert Schaffer 9/25/2018 Reviewed

Reviewer_1_Report_Revision_1 -- Robert Schaffer 12/16/2018 Reviewed

Reviewer_1_Report_Revision_2 -- Robert Schaffer 2/1/2019 Reviewed

Reviewer_2_Report_Original_Submission -- Jian-Feng Mao, Ph.D. 10/16/2018 Reviewed

Supplement_Files.zip
