# Supplementary material for: Chromosome-scale genome assembly of kiwifruit Actinidia eriantha with single-molecule sequencing and chromatin interaction mapping
Source: Gigascience. 2019 Apr 3;8(4):giz027. doi: 10.1093/gigascience/giz027 (PMC6446220; doi:10.1093/gigascience/giz027)
Supplement: GIGA-D-18-00282_Revision_3.pdf [file giz027_giga-d-18-00282_revision_3.pdf]

GigaScience

Chromosome-scale genome assembly of kiwifruit Actinidia eriantha with single-molecule sequencing and chromatin conformation capture

--Manuscript Draft--

**Manuscript Number:**	GIGA-D-18-00282R3
**Full Title:**	Chromosome-scale genome assembly of kiwifruit Actinidia eriantha with single-molecule sequencing and chromatin conformation capture
**Article Type:**	Data Note
**Funding Information:**	National Natural Science Foundation of China (31471157) — Dr. Yongsheng Liu<br>National Science Foundation (IOS-1339287) — Dr. Zhangjun Fei
**Abstract:**	Background: Kiwifruit (Actinidia spp.) is a dioecious plant with fruits containing abundant vitamin C and minerals. A handful of kiwifruit species have been domesticated, among which the A. eriantha is increasingly favored in breeding due to its superior commercial traits. Recently, elite cultivars from A. eriantha have been successfully selected and further studies on their biology and breeding potential require genomic information which is currently unavailable.<br>Findings: Here, we assembled a chromosome-scale genome sequence of A. eriantha cv. 'White' using single-molecular sequencing and chromatin interaction map based scaffolding. The assembly has a total size of 690.6 Mb and an N50 of 21.7 Mb. Approximately 99% of the assembly were in 29 pseudomolecules corresponding to the 29 kiwifruit chromosomes. Forty-three percent of the A. eriantha genome are repetitive sequences, and the non-repetitive part encodes 42,988 protein-coding genes, of which 39,075 have homologues from other plant species or protein domains. The divergence time between A. eriantha and its close relative A. chinensis is estimated to be 3.3 million years, and after diversification, 1,727 and 1,506 gene families are expanded or contracted in A. eriantha, respectively.<br>Conclusions: We provide a high-quality reference genome for kiwifruit A. eriantha. This chromosome-scale genome assembly is substantially better than two published kiwifruit assemblies from A. chinensis in terms of genome contiguity and completeness. The availability of A. eriantha genome provides a valuable resource for facilitating kiwifruit breeding and the studies of kiwifruit biology.
**Corresponding Author:**	Zhangjun Fei<br>Boyce Thompson Institute for Plant Research<br>Ithaca, NY UNITED STATES
**Corresponding Author Secondary Information:**	
**Corresponding Author's Institution:**	Boyce Thompson Institute for Plant Research
**Corresponding Author's Secondary Institution:**	
**First Author:**	Zhangjun Fei
**First Author Secondary Information:**	
**Order of Authors:**	Zhangjun Fei<br>Wei Tang<br>Xuepeng Sun<br>Junyang Yue<br>Xiaofeng Tang<br>Chen Jiao<br>Ying Yang

Powered by Editorial Manager® and ProduXion Manager® from Aries Systems Corporation

	Xiangli Niu
	Min Miao
	Danfeng Zhang
	Shengxiong Huang
	Wei Shi
	Mingzhang Li
	Congbing Fang
	Yongsheng Liu
**Order of Authors Secondary Information:**	
**Response to Reviewers:**	Editor:<br><br>The reviewer has brought up the issue with gene names, and INSDC guidelines for locus tags (see below) they give an example that includes the chromosome number. If used correctly, locus tags can be tracked through different genome builds. See: https://www.ebi.ac.uk/ena/submit/locus-tags or https://www.ncbi.nlm.nih.gov/genomes/locustag/Proposal.pdf<br>We (like the reviewer) recommend following these guidelines. The wording in the INSDC guidelines is " It is preferable to use the same numbering convention for all locus_tags within a project no matter whether the gene is a protein coding gene or structural RNA or from one chromosome or another. "<br><br>We also notice that you have not used the assigned Locus Tag prefix, in the BioProject (PRJNA480681) the locus tag prefix is designated as "DTZ79_" but you have used "Ace" which is too short and likely not unique enough.<br>Response: Thanks. We have updated gene names by replacing the locus tag prefix “Ace” with "DTZ79_". The updated files will be uploaded to GigaDB, and we are also in the process of submitting the genome annotations to NCBI.<br><br>We have read the guidelines carefully and now confirmed that our current gene names are consistent these guidelines. We will follow these guidelines for any future updates.<br><br>The genome assembly you have submitted to the INSDC has quoted accession number QOVS01000000, but that’s not public so we can’t see what they have used there for gene names or locus tags. Can you please also ensure that all the public data is now available.<br>Response: The genome assembly is now public (https://www.ncbi.nlm.nih.gov/nuccore/QOVS00000000.1). We ensure that all the public data is now available. In addition, we have updated “Availability of supporting data” section (Line 293-295) in the revised manuscript.<br><br><br>Reviewer #1:<br><br>This new genome looks like a fantastic improvement to the existing ones in the literature. For the gene models I understand that this is how the community is naming, but I personally have quite strong opinion that this is not the best way. I will let the editor decide what to do on this.<br>Response: Thanks. Please see our responses to Editor’s comments.<br><br>The two examples the authors have given for missing genes are compelling, I would be very happy to if the other 9,998 were like that, as this would represent a much fuller gene lists.<br>Response: Thanks. Again, as stated in our previous response, 90.9% of predicted genes were supported by at least one annotation from seven protein and domain databases searched, and of the remaining genes 71.5% had FPKM value ≥ 1. These suggest that the majority of the predicted genes should be reliable.
**Additional Information:**	

Powered by Editorial Manager® and ProduXion Manager® from Aries Systems Corporation

Question	Response
Are you submitting this manuscript to a special series or article collection?	No
**Experimental design and statistics**<br><br>Full details of the experimental design and statistical methods used should be given in the Methods section, as detailed in our Minimum Standards Reporting Checklist. Information essential to interpreting the data presented should be made available in the figure legends.<br><br>Have you included all the information requested in your manuscript?	Yes
**Resources**<br><br>A description of all resources used, including antibodies, cell lines, animals and software tools, with enough information to allow them to be uniquely identified, should be included in the Methods section. Authors are strongly encouraged to cite Research Resource Identifiers (RRIDs) for antibodies, model organisms and tools, where possible.<br><br>Have you included the information requested as detailed in our Minimum Standards Reporting Checklist?	Yes
**Availability of data and materials**<br><br>All datasets and code on which the conclusions of the paper rely must be either included in your submission or deposited in publicly available repositories (where available and ethically appropriate), referencing such data using a unique identifier in the references and in the "Availability of Data and Materials" section of your manuscript.	Yes

Have you have met the above requirement as detailed in our Minimum Standards Reporting Checklist?	

Manuscript
Click here to access/download;Manuscript;kiwifruit_genome_R3.docx
Click here to view linked References

Chromosome-scale genome assembly of kiwifruit *Actinidia eriantha* with single-molecule sequencing and chromatin interaction mapping

Wei Tang[1,2,3※], Xuepeng Sun[4※], Junyang Yue[1,3※], Xiaofeng Tang[1,3], Chen Jiao[4], Ying Yang[1], Xiangli Niu[1,3], Min Miao[1,3], Danfeng Zhang[3], Shengxiong Huang[3], Wei Shi[3], Mingzhang Li[5], Congbing Fang[1], Zhangjun Fei[4,6]*, Yongsheng Liu[1,2,3]*

[1]School of Horticulture, Anhui Agricultural University, Hefei 230036, China

[2]Ministry of Education Key Laboratory for Bio-resource and Eco-environment, College of Life Science, State Key Laboratory of Hydraulics and Mountain River Engineering, Sichuan University, Chengdu 610064, China

[3]School of Food Science and Engineering, Hefei University of Technology, Hefei 230009, China

[4]Boyce Thompson Institute, Cornell University, Ithaca NY 14853, USA

[5]Sichuan Academy of Natural Resource Sciences, Chengdu 610015, China

[6]U.S. Department of Agriculture-Agricultural Research Service, Robert W. Holley Center for Agriculture and Health, Ithaca, NY 14853, USA

[※]W.T., X.S., J.Y. and F.H. contributed equally to this work

*Correspondence authors: Dr. Zhangjun Fei, email: zf25@cornell.edu or Dr. Yongsheng Liu, email: liuyongsheng1122@hfut.edu.cn

Abstract

Background: Kiwifruit (*Actinidia* spp.) is a dioecious plant with fruits containing abundant vitamin C and minerals. A handful of kiwifruit species have been domesticated, among which the *A. eriantha* is increasingly favored in breeding due to its superior commercial traits. Recently, elite cultivars from *A. eriantha* have been successfully selected and further studies on their biology and breeding potential require genomic information which is currently unavailable.

Findings: Here, we assembled a chromosome-scale genome sequence of *A. eriantha* cv. 'White' using single-molecular sequencing and chromatin interaction map based scaffolding. The assembly has a total size of 690.6 Mb and an N50 of 21.7 Mb. Approximately 99% of the assembly were in 29 pseudomolecules corresponding to the 29 kiwifruit chromosomes. Forty-three percent of the *A. eriantha* genome are repetitive sequences, and the non-repetitive part encodes 42,988 protein-coding genes, of which 39,075 have homologues from other plant species or protein domains. The divergence time between *A. eriantha* and its close relative *A. chinensis* is estimated to be 3.3 million years, and after diversification, 1,727 and 1,506 gene families are expanded or contracted in *A. eriantha*, respectively.

Conclusions: We provide a high-quality reference genome for kiwifruit *A. eriantha*. This chromosome-scale genome assembly is substantially better than two published kiwifruit assemblies from *A. chinensis* in terms of genome contiguity and completeness. The availability of *A. eriantha* genome provides a valuable resource for facilitating kiwifruit breeding and the studies of kiwifruit biology.

Key words: Kiwifruit; *Actinidia eriantha*; Genome assembly; single molecular sequencing; Hi-C

Data description

Introduction

Kiwifruit is well known as the king of fruits due to its remarkably high vitamin C content and abundant minerals [1, 2]. Native to China, kiwifruit belongs to the genus *Actinidia* which contains 54 species and 75 taxa [3]. All species in this genus are perennial, deciduous and dioecious plants with a climbing or scrambling growth habit, and they also have many common morphological features including the characteristic radiating arrangement of styles of female flower and the structure of the fruit [4]. Despite rich germplasm resources in kiwifruit, only a few *Actinidia* species have been domesticated, such as *A. chinensis* var. chinensis, *A. chinensis* var. deliciosa and *A. eriantha*, whose fruit size are close to commercial standard [5-7].

Owing to its strong resistance to *Pseudomonas syringae* pv. *Actinidiae,* long shelf-life, enriched ascorbic acid and peelable skin [7-11], the *A. eriantha* (2n=58) has been favored in kiwifruit breeding. Recently, new cultivars have been selected either from the wild germplasm of *A. eriantha* such as ‘White’ (Fig. 1) or from the interspecific hybridization between *A. eriantha* (♂) and *A. chinensis* (♀) such as ‘Jinyan’ [7, 12]. The ‘White’ has particularly large fruits (96 g on average) with green flesh and favorable flavor and has been widely cultivated in China [7].

Actinidia eriantha has also been used for genetic and genomic studies thanks to its high efficiency in genetic transformation and relatively short phase of juvenility [13]. The flowering and fruiting of *A. eriantha* can be accomplished within two years in green house conditions with a low requirement for winter chilling [13]. In addition, roots of *A. eriantha* which contain many bioactive compounds such as triterpenes and polysaccharides are employed as a traditional Chinese medicine for the treatment of gastric carcinoma, nasopharyngeal carcinoma, breast carcinoma, and hepatitis [12, 14].

Previously, two kiwifruit genomes were published and both were from *A. chinensis* (‘Hongyang’ and ‘Red 5’) [15, 16]. These short-read based assemblies are very fragmented, possibly due to the high complexity and heterozygosity of the kiwifruit genomes as well as technical limitations. Here, we used single-molecular sequencing combined with the high-throughput chromosome conformation capture (Hi-C) technology to assemble the genome of the elite kiwifruit cultivar ‘White’ of *A. eriantha*. The availability of this high-quality chromosome-scale genome sequence not only provides fundamental knowledge regarding kiwifruit biology but also presents a valuable resource for kiwifruit breeding programs.

Sample collection and genome sequencing

Fresh young leaves were collected from a female individual of *A. eriantha* cv. ‘White’. High molecular weight (HMW) genomic DNA was extracted using the CTAB method as described in the protocol (https://www.pacb.com/wp-content/uploads/2015/09/Shared-Protocol-Preparing-Arabidopsis-DNA-for-20-kb-SMRTbell-Libraries.pdf). To construct genomic libraries (SMRTbell libraries) for PacBio long-read sequencing, HMW genomic DNA was sheared into fragments of approximately 20 kb using a Covaris g-Tube (KBiosciences p/n520079), enzymatically repaired and converted to SMRTbell template following the Manufacturer’s instruction (DNA Template Prep Kit 1.0, PacBio p/n 100-259-100). The templates were size-selected using a BluePippin (SageScience, Inc.) to enrich large DNA fragments (> 10 kb) and then sequenced on a PacBio Sequel system. A total of 9 SMRT cells were sequenced, yielding 3,889,480 million reads with a mean and median length of 10,065 and 15,661 bp, respectively, and a total of 39.1 Gb sequences, about 52.5× coverage of the kiwifruit genome with an estimated size of 745.3 Mb based on the flow cytometry analysis (Fig. S1; Table S1).

Three paired-end Illumina libraries with insert sizes of 180, 220 and 500 bp, and seven mate-pair libraries with insert sizes of 3, 4, 5, 8, 10, 15, 17 kb, were prepared using Illumina's Genomic DNA Sample Preparation kit and the Nextera Mate Pair Sample Preparation kit (Illumina, San Diego, CA), respectively. All libraries were sequenced on an Illumina HiSeq 2500 system, which yielded about 80.1 and 97.3 Gb of raw sequence data for paired-end and mate-pair libraries, respectively (Table S1). The raw Illumina paired-end reads were processed to remove duplications, adaptors and low-quality bases using Super-Deduper [17] and Trimmomatic [18] (v0.35), and the mate-pair reads were cleaned using NextClip [19] (v1.3.1) with default parameters. Finally, we obtained 76.6 and 46.2 Gb high-quality cleaned sequences for paired-end and mate-pair libraries, respectively (Table S1).

To construct the Hi-C library, 'White' plants were grown in a greenhouse, and approximately 4~6 grams young leaves were then harvested and subsequently fixed in the formaldehyde (1% v/v) for 10 min at room temperature. The fixation was terminated by adding glycine to a final concentration of 0.125M. The fixed samples were ground into powder in liquid nitrogen and then lysed with the addition of Triton X-100 to a concentration of 1% (v/v). The nuclei were isolated and prepared for Hi-C library construction according to a previously published protocol [20].

Transcriptome sequencing

To improve gene prediction, we generated transcriptome sequences from a pool of mixed tissues of 'White' including root, stem, leaf, flower, and fruits at 7, 30, 60, 90 and 120 days after anthesis. Total RNA was extracted from these tissues using an RNA extraction kit (BIOFIT, China), treated with DNase I and further purified with RNA clean kit (Promega, USA). RNA-Seq libraries were

constructed with the NEBNext® Ultra™ RNA Library Prep Kit (Illumina, USA), and sequenced on an Illumina HiSeq 2500 system using paired-end mode. A total of ~19.5 million raw read pairs were obtained, which were processed with Trimmomatic to remove adaptors. The cleaned reads were assembled *de novo* with Trinity [21] (version 2.4.0). Additionally, we also generated genome-guided assemblies with both Trinity and StringTie [22]. Different transcriptome assemblies were eventually integrated by PASA [23] (version 2.3.3) and used as transcript evidence during gene prediction process. Mapping of RNA-Seq reads to the genome assembly was performed with STAR [24] (version 020201), and read counting on the coding regions was performed with HTSeq [25] (version 0.6.0.).

Chromosome-scale assembly of the A. eriantha genome

Actinidia eriantha is a diecious plant with a heterozygous diploid genome. We estimated the heterozygosity level through the k-mer spectrum analysis with GenomeScope [26] using sequences from the paired-end library with the insert size of 180 bp. The depth distribution of the derived 17-mers clearly showed two separate peaks, based on which we estimated the heterozygosity level of the *A. eriantha* cv. 'White' genome to be 1.21% (Fig. S1).

We then estimated the genome size of *A. eriantha* 'White' using the flow cytometry analysis, with tomato (*Solanum lycopersicum* cv. Ailsa Craig) used as the reference. We also performed flow cytometry analysis on *A. chinensis* cv. Hongyang. Approximately 1 g of young leaves were washed twice in distilled water and then chopped in ice-cold lysis buffer A (10 mmol/L MgSO4, 50 mmol/L KCl, 3.5 mmol/L HEPES pH 7.5, 0.3% (v/v) Triton x-100, 2% PVP 30 (W/V)). After 5 minutes, the crude lysate was passed through a 75-μm pore size nylon mesh to remove large cellular debris. The filtrate (1 ml) was transferred to a 1.5 ml plastic tube and

centrifuged at 1000 rpm for 5 minutes. The supernatant was discard, and the nuclei were then resuspended with lysis buffer B (10 mmol/L $MgSO_4$, 50 mmol/L KCl, 3.5 mmol/L HEPES pH 7.5, 0.3% (v/v) Triton x-100, 0.4 mg/ml Propidium Iodide, 0.04 mg/ml RNase). After 15 minutes, samples were analyzed using a FACS Vantage SE flow cytometer (Becton-Dickinson, San José, USA). Four biological replicates were performed. Based on the 950-Mb genome of tomato, the genome size of 'White' was estimated to be 745.3±7.9 Mb, similar to the genome size of *A. chinensis* (Fig. S1) and consistent with that in a previous report (758 Mb; [27]).

We employed a strategy which took into account the unique advantage of different assemblers to construct the 'White' genome using PacBio long reads. First, PacBio long reads were corrected and assembled using the Canu program [28] (v1.7), which is a modularized pipeline consisting of three primary stages - read correction, trimming and assembly. The Canu-corrected reads were also assembled independently with the wtdbg program (https://github.com/ruanjue/wtdbg), a fast assembler for long noisy reads. Subsequently, the two independent assemblies (one with Canu and another with wtdbg) were merged by Quickmerge [29] (v0.2) to improve the contiguity. The merged assembly was further processed to correct errors using Pilon [30] (version 1.22) with high-quality cleaned Illumina reads from all paired-end and mate-pair libraries representing a total genome coverage of 171× (Table S1). This yielded 2,818,370 nucleotides, 2,495,388 insertions and 1,691,495 deletions being corrected. The resulting final assembled *A. eriantha* cv. 'White' genome contained 4,076 contigs with a N50 length of 539,246 bp and a cumulative size of 690,376,929 bp (Table 1). The contiguity and completeness of this assembly far exceeds that of two published kiwifruit *A. chinensis* genomes (Table 1).

Table 1 Assembly statistics

	A. eriantha	*A. chinensis*	
	**White**	**Hongyang**	**red5**
**Contigs**			
Total contig number (#)	4,076	26,721	39,868
Total contig length (Mb)	690.4	604.2	
Contig N50 (kb)	539.2	58.9	
Contig N90 (kb)	50.7	11.6	
Longest contig length (kb)	3,260.20	423.5	
**Scaffolds**			
Total scaffold number (#)	1,735	7,698	3,887
Total scaffold length (Mb)	690.6	616.1	550.5
Scaffold N50 (kb)	23,583.9	646.8	623.8
Scaffold N90 (kb)	20,112.1	122.7	140.7
Longest scaffold length (Mb)	28.6	3.4	4.43
Anchored to chromosome (Mb/%)	682.4 / 98.84	452.4 / 73.4	547.9 / 98.9
Anchored with order and orientation (Mb/%)	634,4 / 91.90	333.6 / 54.1	

To scaffold the contigs based on chromatin interaction maps inferred from the Hi-C data, we first used HiC-Pro [31] to evaluate and filter the cleaned Hi-C reads. The Hi-C data usually contains a considerable part of invalid interaction read pairs which are non-informative and need to be filtered out beforehand. Among the 51 million read pairs that were uniquely aligned to the *A. eriantha* assembly, 33 million (64.1%) were valid interaction pairs and their insertion size spanned predominantly from dozens to hundreds of kilobases, therefore providing efficient information for scaffolding. As a part of error correction of the assembly, we used valid Hi-C reads to identify misassembled contigs. In principle, a genuine contig should display a continuous Hi-C interaction map whereas the discrete distribution of an interaction map likely indicates a misassembly. We examined the interaction map for each contig and broke 51 that were possibly misassembled. Subsequently, the corrected PacBio assembly was used for scaffolding with the LACHESIS program [32] and parameters “CLUSTER_MIN_RE_SITES=48, CLUSTER_MAX_LINK_DENSITY=2, CLUSTER_NONINFORMATIVE_RATIO=2, ORDER_MIN_N_RES_IN_TRUN=14, ORDER_MIN_N_RES_IN_SHREDS=15”. LACHESIS

assigned 3,666 contigs with a total size of 682,355,494 bp (98.84% of the assembly) into 29 groups corresponding to the 29 kiwifruit chromosomes (Fig. 2 and 3a), among which 634,430,648 bp (91.90%) had defined order and orientation (Table 1 and S2). The final chromosome-scale assembly had a total length of 690,781,529 bp and an N50 of 23,583,865 bp.

Evaluation of the genome assembly

We first evaluated the quality of the assembled *A. eriantha* 'White' genome by mapping Illumina genomic and RNA-Seq reads to the assembly. Reads from the paired-end genomic library (with insert size of 180 bp) had very high mapping rate (98.7%), and the properly paired read mapping rate was 92.0%. For the RNA-Seq reads, 91.7% could be mapped to the genome and 87.1% were uniquely mapped. The high mapping ratio of both genomic and RNA-Seq reads suggest a high quality of the *A. eriantha* 'White' assembly.

We then identified synteny between the *A. eriantha* 'White' assembly and the assembly of *A. chinensis* 'red5' using MUMMER [33] (version 4.0.0beta2). In general, the two assemblies showed a high macro-collinearity, with only a few inconsistencies (Fig. 3b). Detailed check of the major inconsistent regions using genetic maps [34] and mate-pair read alignments confirmed the high quality of the *A. eriantha* 'White' genome assembly, and particularly enabled us to discover that in the 'red5' genome a ~8-Mb region was possibly misassembled into chromosome 23 (Fig. S2).

Repeat annotation

Repeats were annotated following a protocol described in Campbell et al [35]. The customized repeat library was built to include both known and novel repeat families. We first searched the

assembly for miniature inverted transposable elements (MITEs) using MITE-Hunter [36] with default parameters. The long terminal repeat (LTR) retrotransposons were then identified from the *A. eriantha* 'White' genome using LTRharvest and LTRdigest wrapped in the GenomeTools package [37]. The LTR identification pipeline was run iteratively to collect both recent (sequence similarity ≥99%) and old (sequence similarity ≥85%) LTR retrotransposons. Candidates from each run were filtered based on the elements typically encoded by LTR retrotransposons. The default parameters (-minlenltr 100 -maxlenltr 6000 -mindistltr 1500 -maxdistltr 25000 -mintsd 5 -maxtsd 5 -motif tgca) were used in LTR calling according to Campbell *et al*. [35]. An initial repeat masking of *A. eriantha* 'White' genome was performed with the repeat library derived by combining the identified MITEs and LTR transposons. The repeat masked genome was fed to RepeatModeler (http://www.repeatmasker.org/RepeatModeler/) to identify novel repeat families. Finally, all identified repeat sequences were combined and searched against a plant protein database where transposon encoding proteins were excluded. Elements with significant similarity to plant genes were removed. The final repeat library contained 1,670 families, and 526 of them were potentially novel repeat families. We used this species-specific repeat library to mask the *A. eriantha* 'white' genome. Approximately 43.3% of the *A. eriantha* 'White' genome was masked, and the largest family of repeats was LTR transposons (Table S3). Repeat content identified in *A. eriantha* 'White' was much higher than that in *A. chinensis* [e.g. 36% in Hongyang [15]], and this difference may be largely due to the improvement of the repeat region assembly with PacBio long reads. In addition, divergence between the two kiwifruit species could also contribute to this difference.

Prediction and functional annotation of protein-coding genes

Protein-coding genes were predicted from the repeat-masked *A. eriantha* 'White' genome with the MAKER-P program [35] (version 2.31.10), which integrates evidence from protein homology, transcripts and *ab initio* predictions. The homology-based evidence was derived by aligning proteomes from 20 plant species to the 'White' genome assembly with exonerate (v2.26.1; https://www.ebi.ac.uk/about/vertebrate-genomics/software/exonerate). SNAP [38], AUGUSTUS [39] (version 3.3), and GeneMark-ES [40] (version 4.35) were used for *ab initio* gene predictions. RNA-Seq data generated in this study were assembled *de novo* with Trinity and the assembled contigs were aligned to the 'White' genome assembly to provide transcript evidence. Predictions supported by the three different sources of evidence were finally integrated by MAKER-P, which resulted in a total of 52,514 primitive gene models. We then filtered and polished these gene models by two steps. First, we combined our RNA-Seq data with others collected from a previous study [41], and mapped the reads to the 'White' genome using the STAR program [24], and a total of 266 million read pairs were mapped. Based on the mapping, raw count for each predicted gene model was derived and then normalized to CPM (counts per million mapped read pairs). Gene models with ultra-low expression (CPM < 0.1) were less likely to be real genes. Furthermore, we found that these lowly expressed genes had relatively high annotation edit distance (AED) score, an indication of low-confidence as defined by MAKER-P program. Therefore, for gene models with CPM < 0.1, we only kept those containing both pfam domains and homologous sequences in the NCBI nr protein database. After this filtering process 42,751 gene models were kept. Second, the predicted protein-coding genes of kiwifruit *A. chinensis* 'red5' have been manually curated [16], and therefore these gene models should have relatively higher accuracy and could be used to modify *A. eriantha* 'White' gene models whose predictions were not consistently supported by the different types of evidence. To this end, we performed another two *ab initio* predictions using

BRAKER [42] and GeMoMa [43] (version 1.5.2) with ‘red5’ proteome as the sole evidence. These two predictions were compared with the gene models predicted by MAKER-P. Consequently, a total of 237 gene models not predicted by MAKER-P were added and another 415 gene models which had better predictions by BRAKER2 or GeMoMa were used to replace the corresponding gene models predicted by MAKER-P. Finally, we obtained a total of 42,988 protein-coding genes in the *A. eriantha* ‘White’ genome, with a mean coding sequence (CDS) size of 1,004 bp and containing an average of five exons.

The predicted genes were functionally annotated by blasting their protein sequences against TAIR [44], Swiss-Prot [45] and TrEMBL [46] databases with an E-value cutoff of 1e-5. Functional descriptions of the protein hits were assembled with the AHRD program (https://github.com/groupschoof/AHRD) and transferred to *A. eriantha* genes. Protein domains were identified using InterProScan [47] (version 5.29-68.0) by searching the protein sequences against domain databases including PANTHER [48], Pfam [49], SMART [50], and PROSITE [51]. The Gene Ontology (GO) terms were assigned to the *A. eriantha* ‘White’ predicted genes using the Blast2GO program [52] with entries from NCBI protein database and InterProScan. Collectively, 90.9% (N=39,075) of the predicted genes contain at least one annotation from the above databases (Table S4).

Evolutionary and comparative genomic analysis

To infer the divergence time between *A. eriantha* and *A. chinensis*, we identified gene orthology between the two species using MCScanX [53] and calculated synonymous substitution rate (Ks) between each orthologous pair. Three additional species, cultivated tomato (*Solanum lycopersicum*), wild tomato (*S. penellii*) and potato (*S. tuberosum*), were also included in the

analysis. The Ks distribution (Fig. 4a) suggested that the divergence between the two kiwifruit species was earlier than that between the two tomato species. We dated the divergence by assuming a strict molecular clock [54], and the time when *A. eriantha* and *A. chinensis* separated was estimated to be ~3.3 million years ago (Mya), compared to ~1.9 Mya between *S. lycopersicum* and *S. penellii* and ~6.0 Mya between *S. lycopersicum* and *S. tuberosum*. Gene family evolution was analyzed by comparing genomes of *A. eriantha*, *A. chinensis*, *S. lycopersicum*, *S. tuberosum*, *Vitis vinifera*, *Arabidopsis thaliana* and *Oryza sativa*. A total of 17,593 orthogroups were defined by OrthoFinder [55] (version 2.2.6) and among which 1,246 were single-copy gene families (Fig. 4b). The single-copy family genes were aligned and concatenated to build a species phylogenetic tree using IQ-TREE [56] (version 1.5.5) with a best-fitting model (Fig. 4c). Gene family expansion/contraction along the branches of the phylogenic tree was analyzed by CAFÉ [57] (version 4.1). Finally, a total of 1,727 and 1,506 gene families were found apparently expanded and contracted, respectively, in *A. eriantha* (Fig. 4c).

Conclusion

Here, we report a high-quality reference genome of kiwifruit *A. eriantha* cv. 'White'. The assembly from single-molecular sequencing combined with Hi-C scaffolding yielded a highly continuous and complete genome than the two previously published kiwifruit genomes. This genome will provide a valuable source for exploration of genetic basis of unique traits in kiwifruit and also facilitate the studying of sexual determination loci in the dioecious plants.

Availability of supporting data

This Whole Genome Shotgun project has been deposited at DBJ/ENA/GenBank under the accession QOVS00000000. The version described in this paper is version QOVS01000000. Raw sequencing reads have been deposited in the Sequence Read Archive (SRA) database under the accession number SRP155011. The *Actinidia eriantha* ‘White’ genome sequence and the annotation are also available via the *GigaScience* database, GigaDB [58]. Detailed protocols of computational analyses have been deposited in protocols.io [59].

Competing interests

The authors have no competing interests to declare.

Abbreviation

Blast: Basic Local Alignment Search Tool;

CTAB: Cetyl trimethylammonium bromide;

NCBI: National Center for Biotechnology Information;

RNA-Seq: RNA sequencing;

PacBio: Pacific Biosciences;

SMRT: Single Molecule Real-Time;

Mb: megabase;

Gb: gigabase

Acknowledgement

This work was supported by grants from the National Natural Science Foundation of China (31471157 and 31700266), National Foundation for Germplasm Repository of Special

Horticultural Crops in Central Mountain Areas of China (NJF2017-69), National Science Fund for Distinguished Young Scholars (30825030), Key Project from the Government of Sichuan Province (2013NZ0014, 2016NZ0105), Key Project from the Government of Anhui Province (2012AKKG0739;1808085MC57), and the US National Science Foundation (IOS-1339287 and IOS-1539831).

Author contribution

W.T., X.S. and J.Y. contributed equally to this work. W.T., J.Y., X.T., Y.Y., X.N., M.M., D.Z., S.H., W.S., C.F. and M.L. collected plant samples, extracted DNA/RNA, and performed transcriptome sequencing and gene expression analyses; W.T., X.S., J.Y., X.T., C.J., Z.F. and Y.L. performed DNA sequencing, genome assembly, gene annotation, evolution and comparative genomic analyses, and website construction; X.S., W.T., Z.F. and Y.L. wrote and revised the manuscript; Y.L. and Z.F. conceived strategies, designed experiments and managed projects. All authors read and approved the manuscript.

Figure legends

Figure 1. Tree and fruits of *A. eriantha* cv. ‘White’.

Figure 2. Chromatin interaction map of *A. eriantha* derived from Hi-C data. Each group represents an individual chromosome.

Figure 3. Genome of *A. eriantha* and synteny between the two kiwifruit species. (a) Genome landscape of *A. eriantha* cv. 'White'. Track A: gene density, Track B: repeat density, Track C: GC content; all were calculated in a 500-kb window; (b) Genome synteny between *A. eriantha* cv. 'White' and *A. chinensis* 'red5'.

Figure 4. Evolutionary and comparative genomic analyses. (a) Distribution of synonymous substitution rate (Ks) between *A. eriantha* and *A. chinensis*, *S. lycopersicum* and *S. penellii*, and *S. lycopersicum* and *S. tuberosum*; (b) Orthogroups shared by selected species; (c) Species phylogenetic tree and gene family evolution. Numbers on the branch indicate counts of gene family that under either expansion (red) or contraction (green).

Figure S1. Genome characteristics of *A. eriantha and A. chinensis.* (a) Flow cytometry analyses of *A. eriantha* cv. White and *A. chinensis* cv. Hongyang. The main peak (I) indicates G0/G1 cells and the secondary peak (II) represents G2/M cells. (b) Flow cytometry analyses of *A. eriantha* 'White' and *Solanum lycopersicum* cv. Ailsa Craig. Peaks a and b represent the G0/G1 cells of "White" and 'Ailsa Craig', respectively. The genome size of 'White' was estimated to be 745.3±7.9 Mb using 'Ailsa Craig' as the reference. (c) 17-mer distribution of 'White' genomic reads (180bp paired-end library).

Figure S2. Examination of assembly inconsistencies between *A. eriantha* cv. 'White' and *A. chinensis* cv. 'red5'. (a) Validation of genome assembly of 'White' using genetic maps. Horizontal lines within "White" chromosomes indicate gapped regions and lines between chromosomes of two assemblies indicate syntenic regions. (b) A chromosomal segment assembled into the Chr23

in *A. chinenesis* “red5”, is syntenic to the region located at the terminus of Chr19 in *A. eriantha* cv. ‘White’. (c) Snapshots of Illumina mate-pair reads mapped to the junctions of the break point as well as nearby regions supporting the assembly of ‘White’.

Reference

1. Ferguson AR, Ferguson LR. Are kiwifruit really good for you? Acta Hort 2013;**610**:131-138
2. Richardson DP, Ansell J, Drummond LN. The nutritional and health attributes of kiwifruit: a review. Eur J Nutr 2018;1-18.
3. Li JQ, Li XW, Soejarto DD. Actinidiaceae. In: Wu ZY, Raven PH, Hong DY, eds. Flora of China. Beijing: Science Press & St. Louis: Missouri Plant Garden Press; 2007;**12**:pp334-362.
4. Ferguson AR, Huang H. Genetic resources of kiwifruit: domestication and breeding. Hortic Rev. 2007;**33**:1-121.
5. Testolin R. Kiwifruit (Actinidia spp.) in Italy: The history of the industry, international scientific cooperation and recent advances in genetics and breeding. ISHS Acta Horticulturae 2015;47-61.
6. Jo YS, Cho HS, Park MY, Bang GP. Selection of a sweet Actinidia eriantha 'bidan'. ISHS Acta Horticulturae 2017; 253-258.
7. Wu Y, Xie M, Zhang Q et al. Characteristics of 'White': a new easy-peel cultivar of *Actinidia eriantha*. N Z J Crop Hortic Sci 2009;**37**(4):369-373.
8. Atkinson RG, Sharma NN, Hallett IC et al. *Actinidia eriantha*: a parental species for breeding kiwifruit with novel peelability and health attributes. N Z J For Sci 2009;**39**:207-216.
9. Guo R, Landis JB, Moore MJ et al. Development and application of transcriptome-derived microsatellites in *Actinidia eriantha* (Actinidiaceae). Front Plant Sci 2017;**8**:1383.
10. Prakash R, Hallett IC, Wong SF et al. Cell separation in kiwifruit without development of a specialised detachment zone. BMC Plant Biol. 2017;**17**(1):86.
11. Shi ZJ, Zhang HQ, Hui Q et al. The resistance evaluation of different kiwifruit varieties to canker. Acta Agriculturae Zhejiangenis 2014;**26**(3):752-759
12. Zhang D, Gao C, Li R et al. TEOA, a triterpenoid from *Actinidia eriantha*, induces autophagy in SW620 cells via endoplasmic reticulum stress and ROS-dependent mitophagy. Arch Pharm Res 2017;**40(**5):579-591.
13. Wang T, Ran Y, Atkinson RG et al. Transformation of *Actinidia eriantha*: a potential species for functional genomics studies in Actinidia. Plant Cell Rep. 2006;**25**(5):425-431.
14. Wu JG, Ma L, Lin SH et al. Anticancer and anti-angiogenic activities of extract from *Actinidia eriantha* Benth root. J Ethnopharmacol 2017;**203**:1-10.
15. Huang S, Ding J, Deng D et al. Draft genome of the kiwifruit *Actinidia chinensis*. Nat Commun 2013;**4**:2640.
16. Pilkington SM, Crowhurst R, Hilario E et al. A manually annotated *Actinidia chinensis* var. chinensis (kiwifruit) genome highlights the challenges associated with draft genomes and gene prediction in plants. BMC Genomics 2018;**19**(1):257.
17. Petersen KR, Streett DA, Gerritsen AT et al. Super deduper, fast PCR duplicate detection in fastq files. ACM 2015;491-492.
18. Bolger AM, Lohse M, Usadel B. Trimmomatic: a flexible trimmer for Illumina sequence data. Bioinformatics 2014;**30**:2114-20.
19. Leggett RM, Clavijo BJ, Clissold L et al. NextClip: an analysis and read preparation tool for Nextera Long Mate Pair libraries. Bioinformatics 2013;**30**(4):566-568.
20. Rao SS, Huntley MH, Durand NC et al. A 3D map of the human genome at kilobase resolution reveals principles of chromatin looping. Cell 2014;**159**(7):1665-1680.
21. Haas BJ, Papanicolaou A, Yassour M et al. De novo transcript sequence reconstruction from RNA-seq using the Trinity platform for reference generation and analysis. Nat Protoc 2013;**8**(8):1494.
22. Pertea M, Pertea GM, Antonescu CM et al. StringTie enables improved reconstruction of a transcriptome from RNA-seq reads. Nat Biotechnol 2015;**33**(3):290-295.
23. Haas BJ, Delcher AL, Mount SM et al. Improving the Arabidopsis genome annotation using maximal transcript alignment assemblies. Nucleic Acids Res 2003;**31**(19):5654-5666.
24. Dobin A, Davis CA, Schlesinger F et al. STAR: ultrafast universal RNA-seq aligner. Bioinformatics 2013;**29**(1):15-21.

25. Anders S, Pyl PT, Huber W. HTSeq—a Python framework to work with high-throughput sequencing data. Bioinformatics 2015;**31**(2):166-169.
26. Vurture GW, Sedlazeck FJ, Nattestad M et al. GenomeScope: fast reference-free genome profiling from short reads. Bioinformatics 2017;**33**(14):2202-2204.
27. Hopping ME. Flow cytometric analysis of Actinidia species. N Z J Bot 1994;**32**(1):85-93.
28. Koren S, Walenz BP, Berlin K et al. Canu: scalable and accurate long-read assembly via adaptive k-mer weighting and repeat separation. Genome Res 2017;**27**(5):722-736.
29. Chakraborty M, Baldwin-Brown JG, Long AD et al. Contiguous and accurate de novo assembly of metazoan genomes with modest long read coverage. Nucleic Acids Res 2016;**44**(19):e147.
30. Walker BJ, Abeel T, Shea T et al. Pilon: an integrated tool for comprehensive microbial variant detection and genome assembly improvement. PloS One 2014;**9**(11):e112963.
31. Servant N, Varoquaux N, Lajoie BR et al. HiC-Pro: an optimized and flexible pipeline for Hi-C data processing. Genome Biol 2015;**16**(1):259.
32. Burton JN, Adey A, Patwardhan RP et al. Chromosome-scale scaffolding of de novo genome assemblies based on chromatin interactions. Nat Biotechnol 2013;**31**(12):1119.
33. Kurtz S, Phillippy A, Delcher AL et al. Versatile and open software for comparing large genomes. Genome Biol 2004;**5**(2):R12.
34. Zhang Q, Liu C, Liu Y et al. 2015. High-density interspecific genetic maps of kiwifruit and the identification of sex-specific markers. DNA Res 2015;**22**(5):367-375.
35. Campbell M, Law M, Holt C et al. MAKER-P: a tool-kit for the rapid creation, management, and quality control of plant genome annotations. Plant Physiol 2013;**164**(2):513-524.
36. Han Y, Wessler SR. MITE-Hunter: a program for discovering miniature inverted-repeat transposable elements from genomic sequences. Nucleic Acids Res 2010;**38**(22):e199.
37. Gremme G, Steinbiss S, Kurtz S. GenomeTools: a comprehensive software library for efficient processing of structured genome annotations. IEEE/ACM Trans Comput Biol Bioinform 2013;**10**(3):645-656.
38. Korf I. Gene finding in novel genomes. BMC Bioinformatics 2004;**5**(1):59.
39. Stanke M, Keller O, Gunduz I et al. AUGUSTUS: ab initio prediction of alternative transcripts. Nucleic Acids Res. 2006;**34**:W435-W439.
40. Lomsadze A, Ter-Hovhannisyan V, Chernoff YO et al. Gene identification in novel eukaryotic genomes by self-training algorithm. Nucleic Acids Res 2005;**33**(20):6494-6506.
41. Wang Z, Liu Y, Li D et al. Identification of circular RNAs in kiwifruit and their species-specific response to bacterial canker pathogen invasion. Front Plant Sci. 2017;**8**:413.
42. Hoff KJ, Lange S Lomsadze A et al. BRAKER1: Unsupervised RNA-Seq-Based Genome Annotation with GeneMark-ET and AUGUSTUS. Bioinformatics 2016;**32**(5):767-769.
43. Keilwagen J, Wenk M, Erickson JL et al. Using intron position conservation for homology-based gene prediction. Nucleic Acids Res 2016;**44**(9):e89.
44. Rhee SY, Beavis W, Berardini TZ et al. The Arabidopsis Information Resource (TAIR): a model organism database providing a centralized, curated gateway to Arabidopsis biology, research materials and community. Nucleic Acids Res 2003;**31**(1):224-228.
45. Bairoch A, Boeckmann B. The SWISS-PROT protein sequence data bank. Nucleic Acids Res 1991;**19**(Suppl):2247-2249.
46. Bairoch A, Apweiler R. The SWISS-PROT protein sequence data bank and its supplement TrEMBL. Nucleic Acids Res 1997;**25**(1):31-36.
47. Zdobnov EM, Apweiler R. InterProScan–an integration platform for the signature-recognition methods in InterPro. Bioinformatics 2001;**17**(9):847-848.
48. Mi H, Lazareva-Ulitsky B, Loo R et al.The PANTHER database of protein families, subfamilies, functions and pathways. Nucleic Acids Res 2005;**33**(Suppl):D284-D288.
49. Finn RD, Bateman A, Clements J et al. Pfam: the protein families database. Nucleic Acids Res 2014;**42**(Database issue):D222-D230.

50. Schultz J, Copley RR, Doerks T et al. SMART: a web-based tool for the study of genetically mobile domains. Nucleic Acids Res 2000;**28**(1):231-234.
51. Bairoch A. PROSITE: a dictionary of sites and patterns in proteins. Nucleic Acids Res 1991;**19**(Suppl):2241-2245.
52. Conesa A, Götz S. Blast2GO: A comprehensive suite for functional analysis in plant genomics. Int J Plant Genomics 2008;**2008**:619832.
53. Wang Y, Tang H, DeBarry JD et al. MCScanX: a toolkit for detection and evolutionary analysis of gene synteny and collinearity. Nucleic Acids Res 2012;**40**(7):e49.
54. Ossowski S, Schneeberger K, Lucas-Lledó JI et al. The rate and molecular spectrum of spontaneous mutations in *Arabidopsis thaliana*. Science 2010;**327**(5961):92-94.
55. Emms DM, Kelly S. OrthoFinder: solving fundamental biases in whole genome comparisons dramatically improves orthogroup inference accuracy. Genome Biol 2015;**16**(1):157.
56. Nguyen LT, Schmidt HA, von Haeseler A et al. IQ-TREE: a fast and effective stochastic algorithm for estimating maximum-likelihood phylogenies. Mol Biol Evol 2014;**32**(1):268-74.
57. De Bie T, Cristianini N, Demuth JP et al. CAFE: a computational tool for the study of gene family evolution. Bioinformatics 2006;**22**(10):1269-71.
58. Tang W, Sun X, Yue J et al. Supporting data for "Chromosome-scale genome assembly of kiwifruit *Actinidia eriantha* with single-molecule sequencing and chromatin interaction mapping." GigaScience Database 2019.
59. Tang W, Sun X, Yue J et al. Protocols for "Chromosome-scale genome assembly of kiwifruit *Actinidia eriantha* with single-molecule sequencing and chromatin interaction mapping." protocols.io 2019. http://dx.doi.org/10.17504/protocols.io.vgse3we.

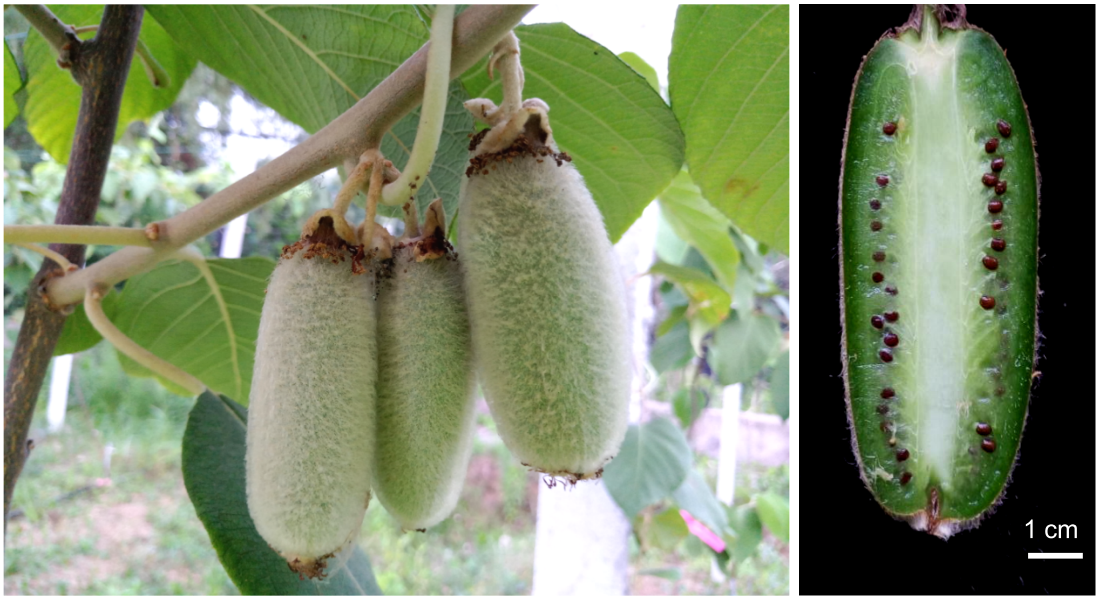

Figure 1

Figure 2 Click here to access/download;Figure;Figure2.pdf

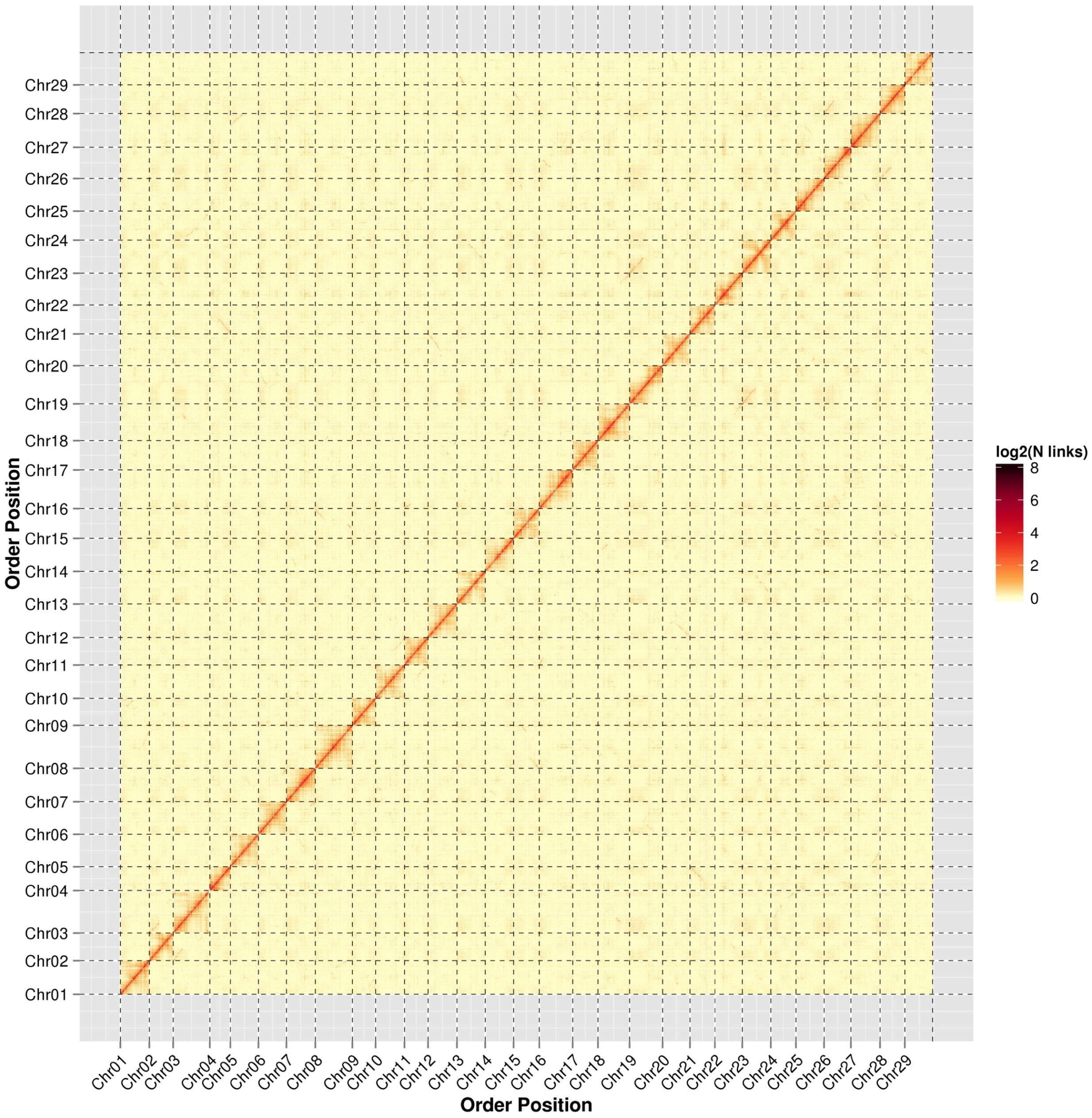

Figure 2.

Figure 3 Click here to access/download;Figure;Figure3.pdf

a

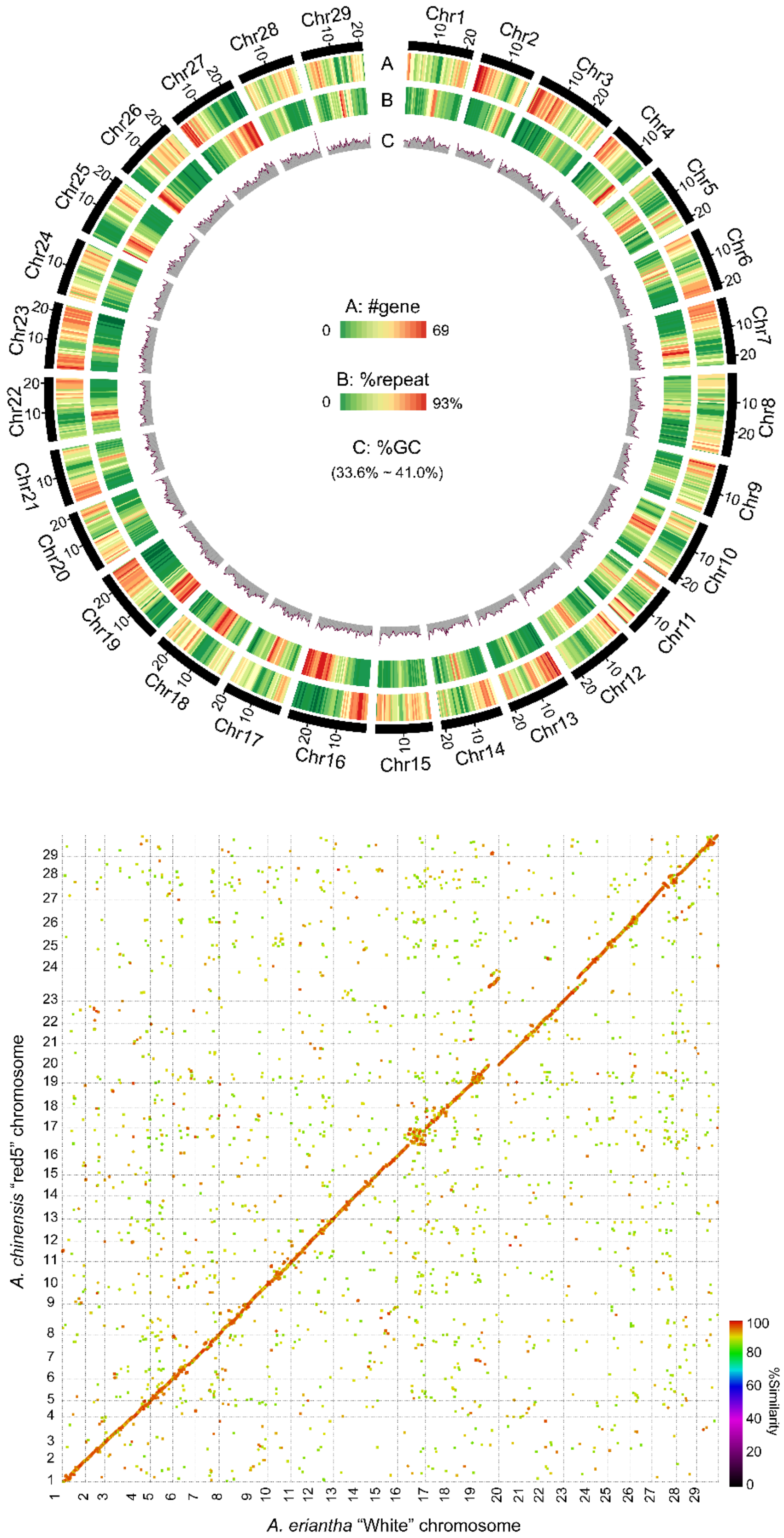

b

Figure 3

Figure 4 Click here to access/download;Figure;Figure4.pdf

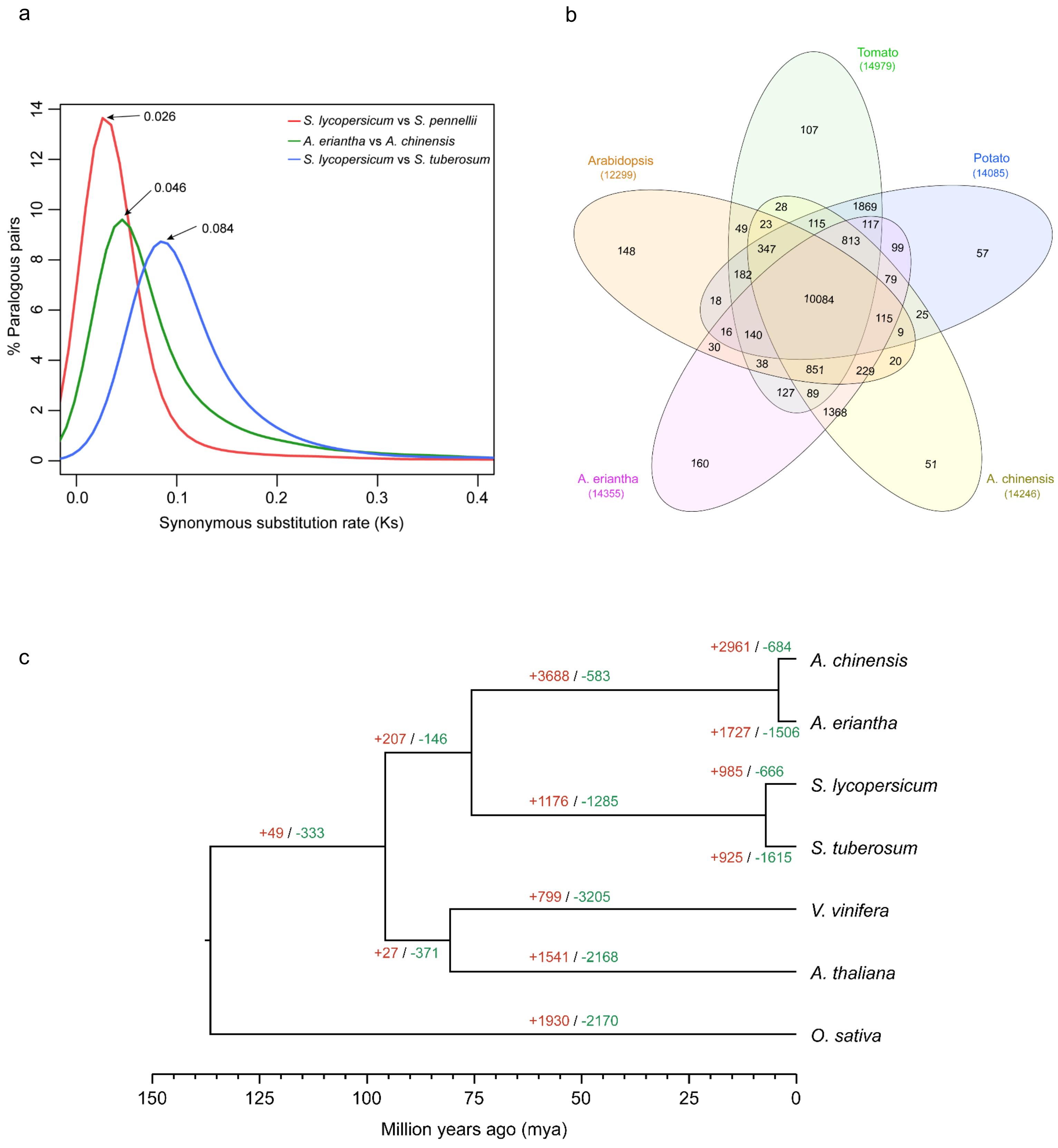

Figure 4

Click here to access/download
Supplementary Material
Supp_Figures.pdf

Supplementary Tables

Click here to access/download
Supplementary Material
Supp_Tables.xlsx

cover letter Click here to access/download;Personal Cover;cover.pdf

February 8, 2019

Dear Dr. Scott Edmunds:

Thank you for your time and effort in handling our manuscript titled "Chromosome-scale genome assembly of kiwifruit *Actinidia eriantha* with single-molecule sequencing and chromatin conformation capture" (manuscript reference number: GIGA-D-18-00282R3).

We have attached our detailed responses to the specific comments of the editor and the reviewer. We hope that we have addressed all the concerns and this revised manuscript is suitable for publication in GigaScience.

Sincerely,

Zhangjun Fei
