# Supplementary material for: Chromosome-scale genome assembly of kiwifruit Actinidia eriantha with single-molecule sequencing and chromatin interaction mapping
Source: Gigascience. 2019 Apr 3;8(4):giz027. doi: 10.1093/gigascience/giz027 (PMC6446220; doi:10.1093/gigascience/giz027)
Supplement: Supplement_Files.zip [file giz027_supplement_files.zip › Supp_Figures.pdf]

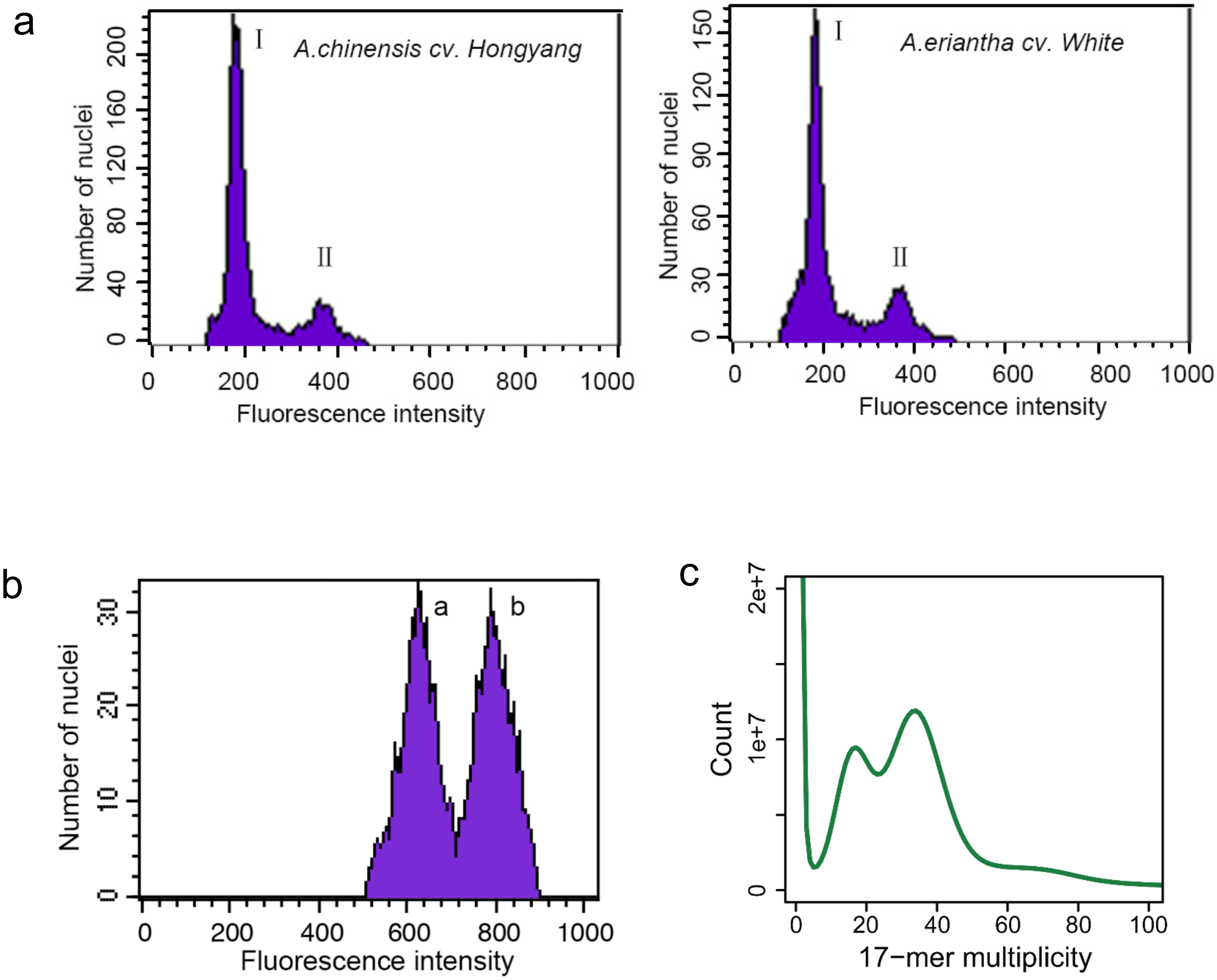

Figure S1. Genome characteristics of *A. eriantha and A. chinensis.* (a) Flow cytometry analyses of *A. eriantha* cv. White and *A. chinensis* cv. Hongyang. The main peak (I) indicates G0/G1 cells and the secondary peak (II) represents G2/M cells. (b) Flow cytometry analyses of *A. eriantha* 'White' and *Solanum lycopersicum* cv. Ailsa Craig. Peaks a and b represent the G0/G1 cells of "White" and 'Ailsa Craig', respectively. The genome size of 'White' was estimated to be 745.3±7.9 Mb using 'Ailsa Craig' as the reference. (c) 17-mer distribution of 'White' genomic reads (180bp paired-end library).

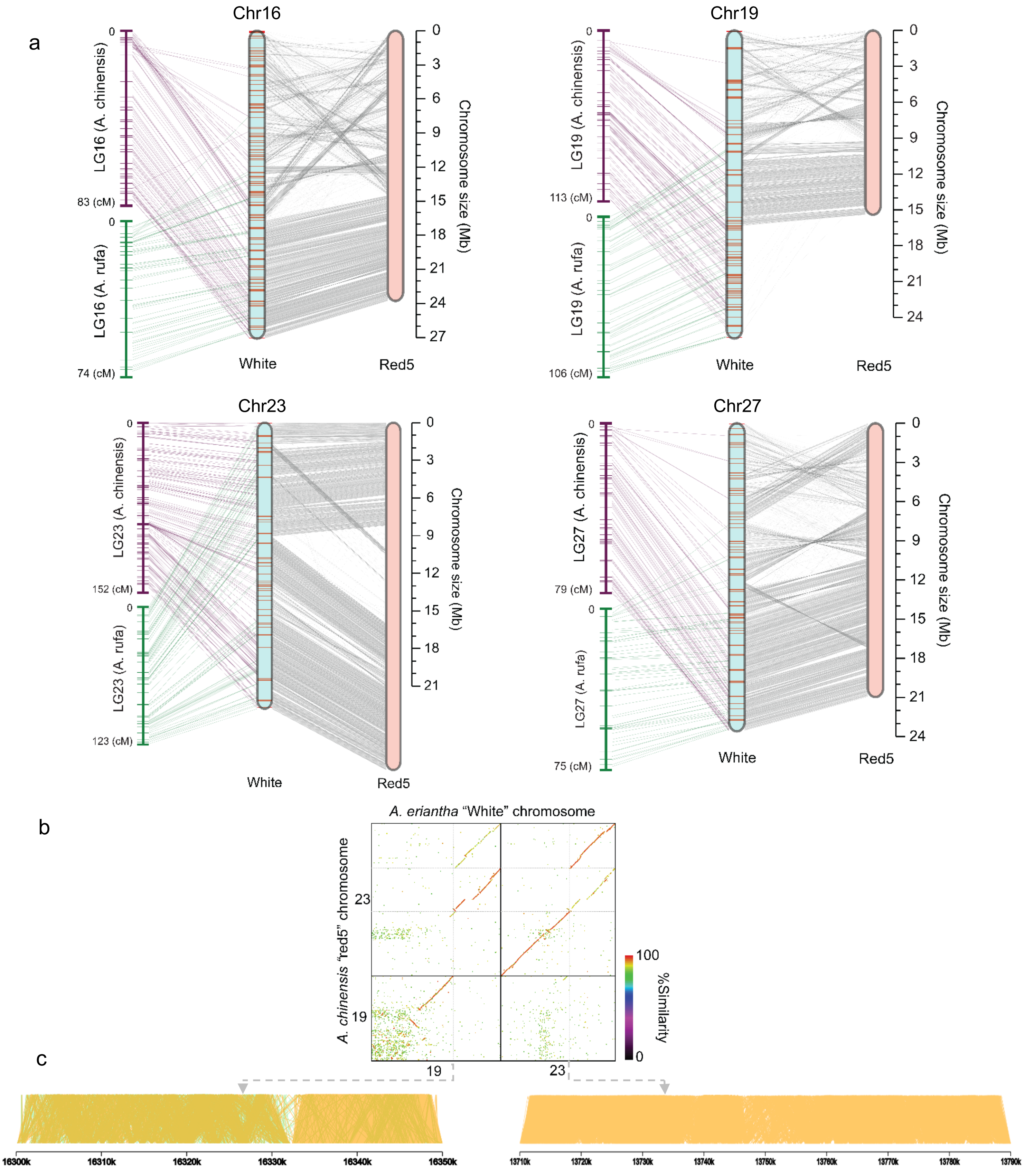

Figure S2. Examination of assembly inconsistencies between *A. eriantha* cv. 'White' and *A. chinensis* cv. 'red5'. (a) Validation of genome assembly of 'White' using genetic maps. Horizontal lines within "White" chromosomes indicate gapped regions and lines between chromosomes of two assemblies indicate syntenic regions. (b) A chromosomal segment assembled into the Chr23 in *A. chinenesis* "red5", is syntenic to the region located at the terminus of Chr19 in *A. eriantha* cv. 'White'. (c) Snapshots of Illumina mate-pair reads mapped to the junctions of the break point as well as nearby regions supporting the assembly of 'White'.
