# Supplementary material for: Chromosome-scale genome assembly of kiwifruit Actinidia eriantha with single-molecule sequencing and chromatin interaction mapping
Source: Gigascience. 2019 Apr 3;8(4):giz027. doi: 10.1093/gigascience/giz027 (PMC6446220; doi:10.1093/gigascience/giz027)
Supplement: Response_to_Reviewer_Comments_Original_Submission.pdf [file giz027_response_to_reviewer_comments_original_submission.pdf]

Author's Response To Reviewer Comments

Close

Note: The response letter was also submitted as a supplementary file.

Editor:
One point raised related to the reproducibility and methodological detail, and one way to address this is to include the protocols in protocols.io.
Response: Thanks. We have uploaded the detailed protocols of computational analyses to protocols.io: http://dx.doi.org/10.17504/protocols.io.vgse3we. This link has been added to the revised manuscript (Line 295-296).

Reviewer #1:
In the paper "Chromosome scale genome assembly of Kiwifruit (Actinidia eriantha) with single molecule sequencing and chromatin conformation capture "the authors present data on a large scale long read assembly of a new kiwifruit species (A. eriantha). This new construction has led to a significant improvement in the amount of sequence that is assembled. Overall the paper is well written with a good quality English.

Major concerns:
1. The authors present the new genome data in the context of the already published data, they state that the new long read construction is more complete and high level of "macro collinearity". Yet the alignment figure (3b) suggests that there are some major differences in the construction (it is hard to tell from the figure which chromosomes) but it appears that chromosome 16 has significant rearrangements, there is a translocation from chr23 to chr19 and a region that is different on chr27. I feel that the wording in the paper does not address these differences.
- Two of these differences have been highlighted in S1. For this paper it needs to be checked to see if this is a species difference (ie a true rearrangement) or whether the red5 or eriantha construction is wrongly assembled. Usually a mapping approach would facilitate this, my recollection was that the original 'hongyang' genome used eriantha interspecific map to anchor the chromosomes. Could this be used?
Response: We thank the reviewer for the suggestion. We have checked the inconsistent regions between the A. eriantha ‘White’ and A. Chinensis ‘red5’ assemblies using the two genetic maps described in Zhang et al. (2015). We found that the genetic maps supported all these regions in the ‘White’ assembly but not the ‘red5’ assembly (Please see Supplementary Figure S2). This confirms the high quality of the ‘White’ assembly and indicates the potential assembly errors in the ‘red5’ assembly. We have modified the related text in the revised manuscript (Line 190-194).

- The use of the word anchor in Table 1 needs to be changed as the eriantha genome was aligned to the chromosomes but the authors did not anchor it with a genetic map (or if they did they have not detailed this).
Response: As indicated in our original manuscript, we used Hi-C chromatin interaction maps to anchor assembled contigs. Using Hi-C data to anchor assembled contigs and the term “anchor” under this context have been widely used in the recently published high-quality genomes.

2. I feel it is important to have some consistency with the naming of gene models found in Actinidia species, as this will ultimately facilitate cross comparisons across species. Indeed this paper is an ideal opportunity to start an Actinidia pan genome gene set. To this end I think it is important that there is a consistent naming convention. In many species the gene locations are given along chromosomes, yet when there is genome divergence, this labelling becomes impossible across species. The manual annotated kiwifruit genome, the genes were given a unique number which bypasses this issue. I was unable to access supplemental data 3 and 4, so apologies if requests below has been done. (maybe some examples in the main body of the paper on the gene models would be of interest).
- We need consistency in the literature. To this end the authors need to name genes in a similar manner to the already published genomes (maybe call them AerXXXXX). And if they could have an orthologue they should be given a number corresponding to the Acc genes already published. New genes should

then be given new numbers
Response: We politely disagree with the reviewer regarding his comments here. In ‘White’, we named the genes based on their chromosome location, which is a nomenclature that has been widely adopted in plant (and animal) genomes, e.g., Arabidopsis, rice, tomato etc. This conventional gene nomenclature (that includes chromosome information) can provide intuitive information such as candidate gene location in QTL mapping, tandemly duplicated genes etc. Actually, this is the nomenclature we should follow, instead of abandon. This is also the reason that in the version 2 of the ‘Hongyang’ genes, we changed gene names by adopting this nomenclature.
Regarding the orthology suggested by the reviewer, we have to point out that this would be very challenging and sometimes confusing due to gene changes in different genomes during their evolution such as gene loss and gain, tandem and segment duplications. This is why the plant community didn’t use the approach suggested by the reviewer, e.g., Arabidopsis (thaliana and lyrata), tomato (lycopersicum and pennellii), rice (indica and japonica).

- The authors need to identify whether the new genes are unique to the eriantha genome and do a quality measure on these to establish whether they are true genes or computational artifacts.
Response: As shown in Table S4, 90.9% of the predicted genes have at least one annotation from the seven databases searched, including NR protein, InterPro, KEGG, GO, Pfam, SMART and PANTHER databases. In addition, 71.5% of the remaining genes (or genes without database support) have FPKM values ≥ 1. Therefore, the majority of the predicted models are true genes instead of computational artifacts.

- It would be good to have an idea whether the genes were new to eriantha or just missed in the A. chinensis manual annotation process.
Response: To address this question, we use orthology analysis (Fig 4) as an example. There are 727 orthogroups contain genes from A. eriantha (980 genes) and other species but not from A. chinensis ‘red5’. Blast search of these genes against red5 genome didn’t result valid alignments, suggesting that these genes could be absent in ‘red5’ or not assembled. In addition, we found 661 A. eriantha genes that were not predicted in red5 but had valid alignments against its genome. Among these genes, 428 were expressed and 188 had annotations. Therefore, we believe at least some of them were not annotated in the red5 genome during the manual process.

Minor concerns
1. P3 Line 24. I am not sure that any Actinidia species have been "domesticated" there is no A. domestica. I would say "commercialised"
Response: Only very few domesticated crops are named with ‘domestica’ (e.g., Malus domestica). No ‘domestica’ does not mean there is no domestication (e.g., rice, maize, tomato…). Actually, “domestication” has been widely used for kiwifruit. For example, please check the paper by Hongwen Huang and Ross Ferguson: Genetic Resources of Kiwifruit: Domestication and Breeding (https://onlinelibrary.wiley.com/doi/10.1002/9780470168011.ch1).

Reviewer #2:
This manuscript provides a high-quality chromosome-scale genome assembly and related resources for an important kiwifruit species. We saw significant improvement in the continuity of genome assembly reported over the previous ones. The genome assembly and related resources will be valuable for kiwifruit breeding and fruti science studies.

The manuscript is generally in good quality, and could be accepted after revision.

############
Comments

1. A general process was reported for genome assembly, quality assessment, annotation and comparision, while details lost, such as settings, important parameters used for a specific software. More information is needed for the whole analytical process described. The availability of the full analytical pipeline is like a rule required by GigaScience, I think.
Response: Please see our response to the editor’s comment.

2. Genome estimatiion on K-mer size is lacking. Other than assembly, K-mer distribution provide another set of information, from which genome size, redundancy, heterozygosity, sequencing error rate could be derived independently to assembly. Please add this.
Response: We agree with reviewer that k-mer statistics provide useful information on genome features.

We have generated a graph for k-mer distribution, and based on this we estimated the heterozygosity level of the 'White' genome. However, due to the heterozygous nature of the genome, our attempts on genome size estimation using k-mer were not successful. Nonetheless, we estimated the genome size of 'White' by flow cytometry analyses. Both k-mer and flow cytometry analysis results have been added in the revised manuscript (Line 125-143).

3. Details are lacking for Evolutionary and comparative analysis. How did you generate a phylogenetic tree? What (software, algorithm, data) you used to generated this tree? How many genes? How did you get those genes? How did you do molecular dating? Divergence time (> 200 mya) seems inconsistency to generally reported divergence time (around 120 mya) for monocots and dicots. It would be better to share the gene alignment used for phylogenetic reconstruction and the generated tree as supplementary files.
Response: Thanks for pointing this out. We have corrected the chronogram and elaborated our methods in the protocol.io (http://dx.doi.org/10.17504/protocols.io.vgse3we). Protein alignments for the phylogeny has been deposited to GigaDB during our initial submission of the manuscript.

4. Citation to published papers or online databases and tools, is lacking for some instances, such as, citations to publised genomes are needed in Table 1. The NCBI nr protein database, TAIR, Swiss-Prot and TrEMBL, PANTHER, Pfam, SMART, and PROSITE databases, they all need citations in a proper way. Please also check supplementary tables, for citation and footnote. Full names are needed to be affiliated with abbreviates, such as SINEs, LINEs, LTRs et al., in the supplementary tables.
Response: Thanks for pointing these out. We have revised these accordingly.

5. Page 9, lines 47-48. "In addition, variations between the two kiwifruit species could also contribute to this difference." "variation" may not be the good word. It is better to use "divergence" to describe the genetic difference among species. Please improve it. I am not native speaker, I am open for different tendency/ideas.
Response: Done. Thanks.

6. How did you generate mapping for genomic and RNA-seq data, when you did "Evaluation of the genome assembly"? "high mapping rates, ranging from 98.6% to 98.8%, and the properly paired read mapping rates were between 76.9% and 90.4%." these ranges are not exact enough for detailed examination. The exact values could be presented in the supplementary tables, such as Table S1, and discussed specifically. Also, it would be interesting to present all the details on the inconsistence revealed by mapping of mate-paired reads to the assembly, in addition to simply the mapping rate values.
Response: We thank the reviewer for pointing this out. We mapped genomic data with BWA with default parameters. Based on the k-mer analysis, we did find that the quality of 220-bp and 500-bp paired-end libraries is not high, therefore sequences from these two libraries were not appropriate for assembly evaluation. Reads from these two libraries were only used for base correction after stringent filtering on the alignments (i.e., uniquely mapped and properly paired). We have recalculated mapping statistics using sequences from the 180-bp library focusing only on anchored chromosomes and the proportion of properly mapped paired reads is 92%. We revised the text accordingly (Line 183-185) Moreover, we have carefully examined inconsistent chromosomal regions with the two genetic maps described in Zhang et al. (2015) (Please see Figure S2). Almost all these regions in the 'White' assembly were supported by the genetic maps.

7. Personally, I would like to know whether the authors could set out to present the functional annotation of Vitamin C biosynthesis pathway or mineral processing pathway, given the pathways are important for kiwifruit community and fruit science. Comparsion among kiwifruit species on genetic composition of such pathways may also interest broader range of readers.
Response: Our manuscript was submitted as a "Data Note". Adding the functional annotation and comparative analysis of Vitamin C biosynthesis pathway or mineral processing pathway could distract the focus of the manuscript. We checked a number of genomes published recently as "Data Note" in GigaScience, none have described specific interesting pathways. However, if the editor and the reviewer still think this is necessary, we are happy to comply.

8. The absence of consecutive line numbers is making harder this review process. Please improved it in the revised version.
Response: Added.

Close
