# Supplementary material for: Chromosome-scale genome assembly of kiwifruit Actinidia eriantha with single-molecule sequencing and chromatin interaction mapping
Source: Gigascience. 2019 Apr 3;8(4):giz027. doi: 10.1093/gigascience/giz027 (PMC6446220; doi:10.1093/gigascience/giz027)
Supplement: Response_to_Reviewer_Comments_Revision_2.pdf [file giz027_response_to_reviewer_comments_revision_2.pdf]

Author's Response To Reviewer Comments

Close

Editor:

The reviewer has brought up the issue with gene names, and INSDC guidelines for locus tags (see below) they give an example that includes the chromosome number. If used correctly, locus tags can be tracked through different genome builds. See:
https://www.ebi.ac.uk/ena/submit/locus-tags or
https://www.ncbi.nlm.nih.gov/genomes/locustag/Proposal.pdf
We (like the reviewer) recommend following these guidelines. The wording in the INSDC guidelines is " It is preferable to use the same numbering convention for all locus_tags within a project no matter whether the gene is a protein coding gene or structural RNA or from one chromosome or another. "

We also notice that you have not used the assigned Locus Tag prefix, in the BioProject (PRJNA480681) the locus tag prefix is designated as "DTZ79_" but you have used "Ace" which is too short and likely not unique enough.
Response: Thanks. We have updated gene names by replacing the locus tag prefix "Ace" with "DTZ79_". The updated files will be uploaded to GigaDB, and we are also in the process of submitting the genome annotations to NCBI.

We have read the guidelines carefully and now confirmed that our current gene names are consistent these guidelines. We will follow these guidelines for any future updates.

The genome assembly you have submitted to the INSDC has quoted accession number QOVS01000000, but that's not public so we can't see what they have used there for gene names or locus tags. Can you please also ensure that all the public data is now available.
Response: The genome assembly is now public (https://www.ncbi.nlm.nih.gov/nuccore/QOVS00000000.1). We ensure that all the public data is now available. In addition, we have updated "Availability of supporting data" section (Line 293-295) in the revised manuscript.

Reviewer #1:

This new genome looks like a fantastic improvement to the existing ones in the literature. For the gene models I understand that this is how the community is naming, but I personally have quite strong opinion that this is not the best way. I will let the editor decide what to do on this.
Response: Thanks. Please see our responses to Editor's comments.

The two examples the authors have given for missing genes are compelling, I would be very happy to if the other 9,998 were like that, as this would represent a much fuller gene lists.
Response: Thanks. Again, as stated in our previous response, 90.9% of predicted genes were supported by at least one annotation from seven protein and domain databases searched, and of the remaining genes 71.5% had FPKM value ≥ 1. These suggest that the majority of the predicted genes should be reliable.

Close
