# Supplementary material for: Chromosome-scale genome assembly of kiwifruit Actinidia eriantha with single-molecule sequencing and chromatin interaction mapping
Source: Gigascience. 2019 Apr 3;8(4):giz027. doi: 10.1093/gigascience/giz027 (PMC6446220; doi:10.1093/gigascience/giz027)
Supplement: Response_to_Reviewer_Comments_Revision_3.pdf [file giz027_response_to_reviewer_comments_revision_3.pdf]

Author's Response To Reviewer Comments

Close

NA

Close
