# Supplementary material for: Chromosome-scale genome assembly of kiwifruit Actinidia eriantha with single-molecule sequencing and chromatin interaction mapping
Source: Gigascience. 2019 Apr 3;8(4):giz027. doi: 10.1093/gigascience/giz027 (PMC6446220; doi:10.1093/gigascience/giz027)
Supplement: Reviewer_1_Report_Original_Submission -- Robert Schaffer — 9/25/2018 Reviewed [file giz027_reviewer_1_report_original_submission.pdf]

Reviewer Report

Title: Chromosome-scale genome assembly of kiwifruit Actinidia eriantha with single-molecule sequencing and chromatin conformation capture

Version: Original Submission **Date:** 9/25/2018

Reviewer name: Robert Schaffer

Reviewer Comments to Author:

In the paper "Chromosome scale genome assembly of Kiwifruit (Actinidia eriantha) with single molecule sequencing and chromatin conformation capture" the authors present data on a large scale long read assembly of a new kiwifruit species (A. eriantha). This new construction has led to a significant improvement in the amount of sequence that is assembled. Overall the paper is well written with a good quality English.

Major concerns:

1. The authors present the new genome data in the context of the already published data, they state that the new long read construction is more complete and high level of "macro collinearity". Yet the alignment figure (3b) suggests that there are some major differences in the construction (it is hard to tell from the figure which chromosomes) but it appears that chromosome 16 has significant rearrangements, there is a translocation from chr23 to chr19 and a region that is different on chr27. I feel that the wording in the paper does not address these differences.

- Two of these differences have been highlighted in S1. For this paper it needs to be checked to see if this is a species difference (ie a true rearrangement) or whether the red5 or eriantha construction is wrongly assembled. Usually a mapping approach would facilitate this, my recollection was that the original 'hongyang' genome used Eriantha interspecific map to anchor the chromosomes. Could this be used?
- The use of the word anchor in Table 1 needs to be changed as the eriantha genome was aligned to the chromosomes but the authors did not anchor it with a genetic map (or if they did they have not detailed this.

2. I feel it is important to have some consistency with the naming of gene models found in Actinidia species, as this will ultimately facilitate cross comparisons across species. Indeed this paper is an ideal opportunity to start an Actinidia pan genome gene set. To this end I think it is important that there is a consistent naming convention. In many species the gene locations are given along chromosomes, yet when there is genome divergence, this labelling becomes impossible across species. The manual annotated kiwifruit genome, the genes were given a unique number which bypasses this issue. I was unable to access supplemental data 3 and 4, so apologies if requests below has been done. (maybe some examples in the main body of the paper on the gene models would be of interest).

- We need consistency in the literature. To this end the authors need to name genes in a similar manner to the already published genomes (maybe call them AerXXXXX). And if they could have an orthologue they should be given a number corresponding to the Acc genes already published. New genes should then be given new numbers

- The authors need to identify whether the new genes are unique to the eriantha genome and do a quality measure on these to establish whether they are true genes or computational artifacts.
- It would be good to have an idea whether the genes were new to eriantha or just missed in the A. chinensis manual annotation process.

Minor concerns

1. P3 Line 24. I am not sure that any Actinidia species have been "domesticated" there is no A. domestica. I would say "commercialised"

Level of Interest

Please indicate how interesting you found the manuscript: Choose an item.

Quality of Written English

Please indicate the quality of language in the manuscript: Choose an item.

Declaration of Competing Interests

Please complete a declaration of competing interests, considering the following questions:

- Have you in the past five years received reimbursements, fees, funding, or salary from an organisation that may in any way gain or lose financially from the publication of this manuscript, either now or in the future?
- Do you hold any stocks or shares in an organisation that may in any way gain or lose financially from the publication of this manuscript, either now or in the future?
- Do you hold or are you currently applying for any patents relating to the content of the manuscript?
- Have you received reimbursements, fees, funding, or salary from an organization that holds or has applied for patents relating to the content of the manuscript?
- Do you have any other financial competing interests?
- Do you have any non-financial competing interests in relation to this paper?

If you can answer no to all of the above, write 'I declare that I have no competing interests' below. If your reply is yes to any, please give details below.

'I declare that I have no competing interests'

I agree to the open peer review policy of the journal. I understand that my name will be included on my report to the authors and, if the manuscript is accepted for publication, my named report including any attachments I upload will be posted on the website along with the authors' responses. I agree for my report to be made available under an Open Access Creative Commons CC-BY license (http://creativecommons.org/licenses/by/4.0/). I understand that any comments which I do not wish to

be included in my named report can be included as confidential comments to the editors, which will not be published.

I agree to the open peer review policy of the journal

To further support our reviewers, we have joined with Publons, where you can gain additional credit to further highlight your hard work (see: https://publons.com/journal/530/gigascience). On publication of this paper, your review will be automatically added to Publons, you can then choose whether or not to claim your Publons credit. I understand this statement.

Yes Choose an item.
