# Supplementary material for: Chromosome-scale genome assembly of kiwifruit Actinidia eriantha with single-molecule sequencing and chromatin interaction mapping
Source: Gigascience. 2019 Apr 3;8(4):giz027. doi: 10.1093/gigascience/giz027 (PMC6446220; doi:10.1093/gigascience/giz027)
Supplement: Reviewer_1_Report_Revision_1 -- Robert Schaffer — 12/16/2018 Reviewed [file giz027_reviewer_1_report_revision_1.pdf]

Reviewer Report

Title: Chromosome-scale genome assembly of kiwifruit Actinidia eriantha with single-molecule sequencing and chromatin conformation capture

Version: Revision 1 **Date:** 12/16/2018

Reviewer name: Robert Schaffer

Reviewer Comments to Author:

While the paper has been improved I am disappointed by the responses from the authors especially regarding the gene models
Firstly on the genome construction side. it appears that the anomolies that the authors have spotted are in recombination cold spots (Figure s2) and following the lines, the genetic map does not alway correlate with their assembly? There appears to be an inversion in the construction on ch16 and 19. This would be nice to be resolved.
For gene naming I agree if the quality of the genome was to the standard of the arabidopsis, rice or tomato genome then I it would maybe appropriate to name using genome location.
The 3 reasons for NOT giving names by chromosomal location are:
1) Gene names need to be locked for subsequent functional analysis. In Arabidopsis they changed twice and this was a disaster for publications between 2000 and 2003.
2) the genome arrangement will change in the future and therefore it is too early to give a chromosome position in the name. We are not in a position to give gene names based on chromosome location.
3) Chromosome 30 is non existant (I have not managed to find the gene list, but if it is there then it makes the whole gene naming in this paper irrelevant)
I accept that the orthology maybe a challenge to implement.
FOr the 10,000 new genes I am also concerned that the authors are relying on peptide lists from non verified genes.. I have seen many genome locations that have random RNAseq reads aligned (often in a little repetative section that are meaningless and computer generated models are pushed into the creation of a "gene" where there is not a gene, that goes beyond the areas of RNA seq reads. Did the authors check that these genes have good RNA seq reads accross the whole predicted gene? I would be interested to see some validation of the new gene models. Some examples would be nice rather than assertions by the authors, as this would be a significant improvement to our current understanding.

Level of Interest

Please indicate how interesting you found the manuscript: Choose an item.

Quality of Written English

Please indicate the quality of language in the manuscript: Choose an item.

Declaration of Competing Interests

Please complete a declaration of competing interests, considering the following questions:

- Have you in the past five years received reimbursements, fees, funding, or salary from an organisation that may in any way gain or lose financially from the publication of this manuscript, either now or in the future?
- Do you hold any stocks or shares in an organisation that may in any way gain or lose financially from the publication of this manuscript, either now or in the future?
- Do you hold or are you currently applying for any patents relating to the content of the manuscript?
- Have you received reimbursements, fees, funding, or salary from an organization that holds or has applied for patents relating to the content of the manuscript?
- Do you have any other financial competing interests?
- Do you have any non-financial competing interests in relation to this paper?

If you can answer no to all of the above, write 'I declare that I have no competing interests' below. If your reply is yes to any, please give details below.

'I declare that I have no competing interests'

I agree to the open peer review policy of the journal. I understand that my name will be included on my report to the authors and, if the manuscript is accepted for publication, my named report including any attachments I upload will be posted on the website along with the authors' responses. I agree for my report to be made available under an Open Access Creative Commons CC-BY license (http://creativecommons.org/licenses/by/4.0/). I understand that any comments which I do not wish to be included in my named report can be included as confidential comments to the editors, which will not be published.

I agree to the open peer review policy of the journal

To further support our reviewers, we have joined with Publons, where you can gain additional credit to further highlight your hard work (see: https://publons.com/journal/530/gigascience). On publication of this paper, your review will be automatically added to Publons, you can then choose whether or not to claim your Publons credit. I understand this statement.

Yes Choose an item.
