# Supplementary material for: Chromosome-scale genome assembly of kiwifruit Actinidia eriantha with single-molecule sequencing and chromatin interaction mapping
Source: Gigascience. 2019 Apr 3;8(4):giz027. doi: 10.1093/gigascience/giz027 (PMC6446220; doi:10.1093/gigascience/giz027)
Supplement: Reviewer_2_Report_Original_Submission -- Jian-Feng Mao, Ph.D. — 10/16/2018 Reviewed [file giz027_reviewer_2_report_original_submission.pdf]

Reviewer Report

Title: Chromosome-scale genome assembly of kiwifruit Actinidia eriantha with single-molecule sequencing and chromatin conformation capture

Version: Original Submission **Date:** 10/16/2018

Reviewer name: Jian-Feng Mao, Ph.D.

Reviewer Comments to Author:

This manuscript provides a high-quality chromosome-scale genome assembly and related resources for an important kiwifruit species. We saw significant improvement in the continuity of genome assembly reported over the previous ones. The genome assembly and related resources will be valuable for kiwifruit breeding and fruti science studies.
The manuscript is generally in good quality, and could be accepted after revision.
############
Comments
1. A general process was reported for genome assembly, quality assessment, annotation and comparision, while details lost, such as settings, important parameters used for a specific software. More information is needed for the whole analytical process described. The availability of the full analytical pipeline is like a rule required by GigaScience, I think.
2. Genome estimatiion on K-mer size is lacking. Other than assembly, K-mer distribution provide another set of information, from which genome size, redundancy, heterozygosity, sequencing error rate could be derived independently to assembly. Please add this.
3. Details are lacking for Evolutionary and comparative analysis. How did you generate a phylogenetic tree? What (software, algorithm, data) you used to generated this tree? How many genes? How did you get those genes? How did you do molecular dating? Divergence time (> 200 mya) seems inconsistency to generally reported divergence time (around 120 mya) for monocots and dicots. It would be better to share the gene alignment used for phylogenetic reconstruction and the generated tree as supplementary files.
4. Citation to published papers or online databases and tools, is lacking for some instances, such as, citations to publised genomes are needed in Table 1. The NCBI nr protein database, TAIR, Swiss-Prot and TrEMBL, PANTHER, Pfam, SMART, and PROSITE databases, they all need citations in a proper way. Please also check supplementary tables, for citation and footnote. Full names are needed to be affiliated with abbreviates, such as SINEs, LINEs, LTRs et al., in the supplementary tables.
5. Page 9, lines 47-48. "In addition, variations between the two kiwifruit species could also contribute to this difference." "variation" may not be the good word. It is better to use "divergence" to describe the genetic difference among species. Please improve it. I am not native speaker, I am open for different tendency/ideas.
6. How did you generate mapping for genomic and RNA-seq data, when you did "Evaluation of the genome assembly"? "high mapping rates, ranging from 98.6% to 98.8%, and the properly paired read mapping rates were between 76.9% and 90.4%." these ranges are not exact enough for detailed

examination. The exact values could be presented in the supplementary tables, such as Table S1, and discussed specifically. Also, it would be interesting to present all the details on the inconsistence revealed by mapping of mate-paired reads to the assembly, in addition to simply the mapping rate values.

7. Personally, I would like to know whether the authors could set out to present the functional annotation of Vitamin C biosynthesis pathway or mineral processing pathway, given the pathways are important for kiwifruit community and fruit science. Comparsion among kiwifruit species on genetic composition of such pathways may also interest broader range of readers.

8. The absence of consecutive line numbers is making harder this review process. Please improved it in the revised version.

Level of Interest

Please indicate how interesting you found the manuscript: Choose an item.

Quality of Written English

Please indicate the quality of language in the manuscript: Choose an item.

Declaration of Competing Interests

Please complete a declaration of competing interests, considering the following questions:

- Have you in the past five years received reimbursements, fees, funding, or salary from an organisation that may in any way gain or lose financially from the publication of this manuscript, either now or in the future?
- Do you hold any stocks or shares in an organisation that may in any way gain or lose financially from the publication of this manuscript, either now or in the future?
- Do you hold or are you currently applying for any patents relating to the content of the manuscript?
- Have you received reimbursements, fees, funding, or salary from an organization that holds or has applied for patents relating to the content of the manuscript?
- Do you have any other financial competing interests?
- Do you have any non-financial competing interests in relation to this paper?

If you can answer no to all of the above, write 'I declare that I have no competing interests' below. If your reply is yes to any, please give details below.

I declare that I have no competing interests.

I agree to the open peer review policy of the journal. I understand that my name will be included on my report to the authors and, if the manuscript is accepted for publication, my named report including any

attachments I upload will be posted on the website along with the authors' responses. I agree for my report to be made available under an Open Access Creative Commons CC-BY license (http://creativecommons.org/licenses/by/4.0/). I understand that any comments which I do not wish to be included in my named report can be included as confidential comments to the editors, which will not be published.

I agree to the open peer review policy of the journal

To further support our reviewers, we have joined with Publons, where you can gain additional credit to further highlight your hard work (see: https://publons.com/journal/530/gigascience). On publication of this paper, your review will be automatically added to Publons, you can then choose whether or not to claim your Publons credit. I understand this statement.

Yes Choose an item.
